# Experimental and Computational Studies Unraveling the Peculiarity of Enolizable Oxoesters in the Organocatalyzed Mannich-Type Addition to Cyclic *N*-Acyl Iminium Ions

**DOI:** 10.3390/molecules25081903

**Published:** 2020-04-20

**Authors:** Andrea Menichetti, Sebastiano Di Pietro, Valeria Di Bussolo, Lucilla Favero, Mauro Pineschi

**Affiliations:** 1 Department of Pharmacy, University of Pisa, via Bonanno 33, 56126 Pisa, Italy; a.menichetti90@gmail.com (A.M.); sebastiano.dipietro@unipi.it (S.D.P.); 2 Department of Chemistry and Industrial Chemistry, University of Pisa, via Moruzzi 3, 56124 Pisa, Italy; valeria.dibussolo@unipi.it

**Keywords:** *N*,*O*-acetals, organocatalysis, oxoesters, Mannich reactions, DFT study, heteroaryl lactones

## Abstract

γ− and δ-Oxoesters are easily available starting materials that have been sparingly used in some organocatalyzed reactions proceeding with a high enantioselectivity. In our experimentation we found that the use of these compounds as the enolizable (nucleophilic) component in organocatalyzed Mannich-type reactions using in situ-generated cyclic *N*-acyl iminium ions gave low diastereoselectivity and low to moderate values of enantioselectivity. This significant drop of facial selectivity with respect to simple aliphatic aldehydes has been rationalized by means of density functional theory (DFT) calculations.

## 1. Introduction

**Figure 1:**
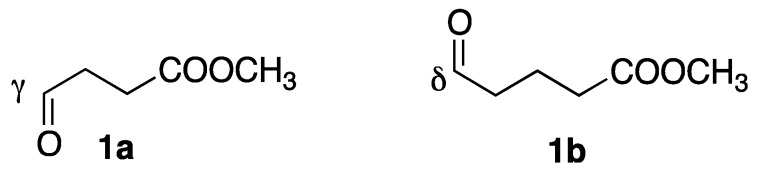
Oxoesters examined in this study.

Difunctionalized compounds such δ- and γ-oxoesters **1a,b** have been widely used in organic synthesis due to their rich chemistry (Figure 1). In a seminal example, the exploitation of the reactivity of aldehyde functionality of alkyl 5-oxopentanoates has been exploited in the synthesis of d,l-biotin [1].

**Scheme 1:**
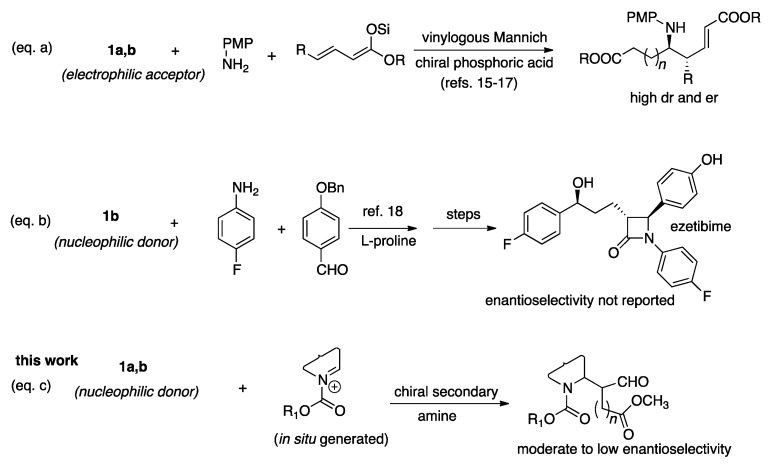
Use of oxoesters **1a,b** in organocatalyzed Mannich-type reactions.

Alkyl 5-oxopentanoates have also been frequently used in Horner-Wadsworth-Emmons (HEW)-olefinations to give a variety of biologically active natural products [2,3,4,5,6,7,8,9]. Notwithstanding the increasing importance of organocatalysis in modern synthetic organic chemistry and the large variety of reactions described, the use of oxoesters in organocatalyzed reactions has not been considered to a large extent. For example, δ- and γ-oxoesters have been used as the enolizable (nucleophilic) component in secondary amine-organocatalyzed Michael-type [10,11,12,13] and aldol reactions [14], all featuring high enantioselectivity [>90% enantiomeric excess (ee)]. Oxoesters **1a,b** have also been employed in three-component vinylogous Mannich (VM) reactions as the *electrophilic acceptors* upon reaction with an aromatic amine followed by several steps to obtain the indolizidine and quinolizidine frameworks with very high enantio- and diastereoselectivity (Scheme 1, eq. a) [15,16,17].

To the best of our knowledge, we are aware of only one example featuring a Mannich reaction of oxoesters **1a,b**, in which these compounds behave as *nucleophilic donor*. In this example, δ-oxoester **1b** was used in an L-proline-catalyzed asymmetric three-component Mannich reaction during the synthesis of the β-lactam skeleton of ezetimibe without any determination of the enantiomeric purity of the corresponding product (Scheme 1, eq. b) [18].

This paucity of interest about the use of enolizable oxoesters as nucleophilic partner in Mannich-type reaction prompted us to explore this reactivity using in situ-generated *N*-acyl iminium ions as the electrophilic component. Dearomative Mannich-type reactions have recently been reported by us and other groups using activated quinolinium ions as the electrophilic acceptor, both using synergistic cooperative catalysis starting from *N,O*-acetals [19,20,21,22], or directly from quinolines [23]. Moreover, the frequent occurrence of 1-substituted-1,2,3,4-tetrahydroisoquinoline ring systems has stimulated considerable interest in their asymmetric synthesis by the use of metal- and organocatalysis [24]. Major advances using an organocatalytic approach have been developed by Cozzi et al., by enantioselective addition of aldehydes to isoquinolinium carbamates [25], and by Liu et al., using a cross-dehydrogenative coupling of carbamoyl isoquinolines with aldehydes [26].

## 2. Results and Discussion

### 2.1. Metal-Organocatalyzed Mannich Reactions with Quinoline N,O-Acetals

**Scheme 2:**
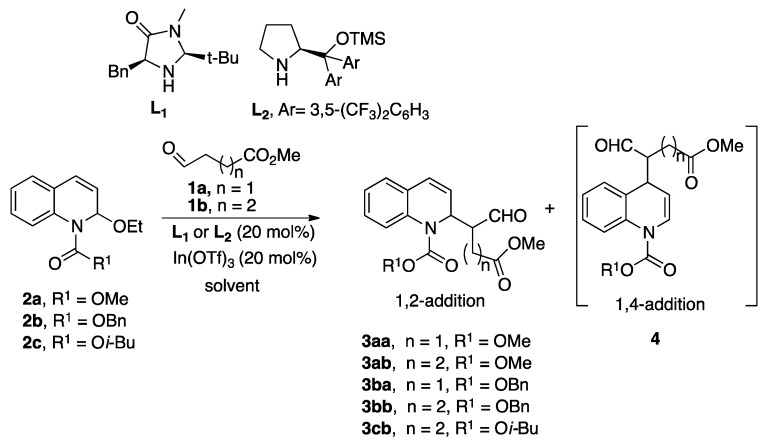
*N*-acyl Mannich reaction of oxoesters **1a,b** with quinoline *N,O*-acetals **2a**–**c**.

Acyl Mannich-type reactions, by the combination of simple aminocatalysts and metal catalysts, have been recently developed with aliphatic and α,β-unsaturated aldehydes by our research group [20,27,28]. Since our experience in this field, we have always noticed that MacMillan catalyst **L_1_** and Hayashi–Jørgensen catalyst **L_2_** showed the best performances in tetrahydrofuran (THF) and toluene, respectively (Scheme 2). In our studies using cooperative dual catalysis we had observed that the combination with different Lewis or Bronsted acids influenced to some extent the reactivity and stereoselectivity depending on the substrate used. As the reactions with aliphatic aldehydes gave similar and optimal results in term of rate, region, and stereocontrol when indium triflate [In(OTf)_3_] was employed with both imidazolidinone catalyst **L_1_** and prolinol organocatalyst **L_2_** this salt was used as catalysts for the reaction of aliphatic oxoesters **1a,b**.

The reaction of oxoesters **1a,b** with quinoline-derived *N,O*-acetal **2a** catalyzed by the combination of organocatalyst **L_1_** and **L_2_** and In(OTf)_3_ occurred with the formation of the corresponding 1,2-addition products of type **3**. Unfortunately, all compounds of type **3** and **4** were obtained as diastereoisomeric mixtures, inseparable by chromatography on silica gel (Table 1, entries 1–6). 

A similar trend was observed with carbobenzyloxy (Cbz)-protected *N,O*-acetal **2b**. With this substrate, the determination of the enantioselectivity was not possible due to lack of chromatographic separation on chiral stationary phases (Table 1, entries 7–10). We also noticed that the poor diastereoselectivity of the reaction cannot be increased by the use of a bulkier protecting group (Table 1, entry 11). The *syn*/*anti* ratio for compounds of type **3** was given on the basis of the integration of aldehydic signals, in close analogy with the assignments made with simple aliphatic aldehydes. The presence of 1,4-adducts of type **4** was determined by ^1^H NMR by their characteristic signals, but their low amounts in the crude mixture in many cases did not allow any separation in a pure state. It should be noted that the enantioselectivities obtained for 1,2-adducts of type **3** were uniformly much lower than those obtained using simple aliphatic aldehydes with substrates **2a,b** in similar reaction conditions [19,20,21]. Also, using a lower reaction temperature (−20 °C) the enantioselectivities were only marginally influenced (Table 1, entries 3,4).

### 2.2. Metal-Organocatalyzed Mannich Reactions with Isoquinoline N,O-Acetals

**Scheme 3:**
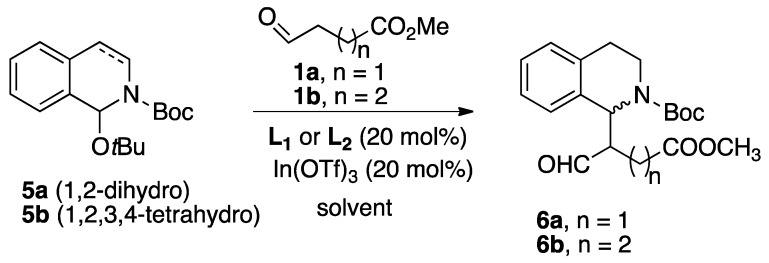
*N*-acyl Mannich reaction of oxoesters **1a,b** with isoquinoline *N,O*-acetals **5a,b**.

To avoid any regioselectivity issue, we also examined dihydroisoquinoline *N,O*-acetal derivatives **5a** (Scheme 3). In accordance with previous data reported by Liu et al. for a related substrate [19], the synergistic metal-organocatalyzed dearomatization-alkylation reaction of **5a** with oxoesters **1a,b** was not possible (<10% conversion, Table 2, entry 1).

On the other hand, tetrahydroquinoline *N,O*-acetal **5b**, easily prepared from the corresponding 3,4-dihydroquinoline using a reported procedure [29], showed to be a suitable reaction partner in our reaction conditions (entries 2–5). The corresponding 1-substituted tetrahydroisoquinolines **6a,b** containing two stereogenic centers were obtained with a low diastereoselectivity and up 70% ee for one diastereoisomer.

### 2.3. Reductive Cyclization to Heteroaryl Lactones

**Scheme 4:**
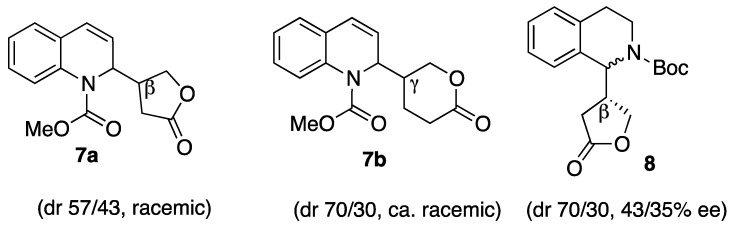
Heteroaryl-substituted lactones obtained.

After chromatographic purification, the reduction of the newly obtained oxoesters **3aa**, **3ab,** and **6a** with NaBH_4_ *gave spontaneously* new heterocyclic δ- and γ-lactones **7a,b** and **8** as the major products (Scheme 4). Functionalized γ- and δ-lactones are valuable compounds found in a large variety of natural and synthetic biologically active substances and many efforts have been devoted to their synthesis in non-racemic form [30,31]. In particular, chiral γ-butyrolactones having an aryl group at β-stereocenter are of considerable importance in themselves and as key precursors to the synthesis of γ-aminobutyric acid derivatives [32,33,34]. While the enantioselective introduction of the aryl moiety at the β-position has been accomplished by asymmetric conjugate addition [35,36,37], or Heck–Matsuda coupling [38], the introduction of nitrogen heterocycles remains challenging [39]. Furthermore, to the best of our knowledge, the introduction of a heteroaryl moiety into the γ-position of a δ-lactone also in a racemic fashion is unprecedented. Our approach gives the possibility of a streamlined introduction of a heteroaryl moiety into the γ-position of a δ-lactone and into the β-position of a γ-lactone. However, the poor stereocontrol obtained for their formation, especially for dihydroquinoline derivatives, points to an unwanted epimerization during the reduction/lactonization process (Scheme 4).

### 2.4. Computational Data

**Figure 2:**
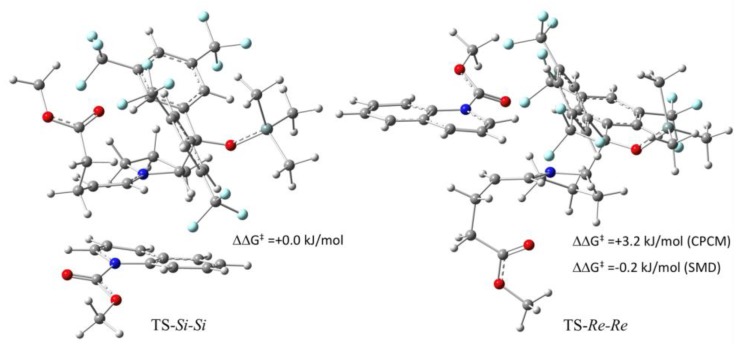
Transition states (TS) leading to (*R*,*R*)-**3ab** and (*S*,*S*)-**3ab**, respectively, using organocatalyst **L_2_**.

As high enantioselectivities (>85% ee) were generally observed by other authors using oxoesters **1a,b** as the nucleophilic component in aldol and Michael reactions [10,11,12,13,14], we initially surmised an epimerization of the exocyclic stereocenter in our Mannich-type reaction. However, a closer examination of the reaction of *N,O*-acetal **2a** with oxoester **1b** in the presence of **L_1_** and **L_2_** showed not significant variations during time. Even after 5 min the product distribution was roughly the same as reported in Table 1 and did not have substantial changes during time, thus pointing to a stereochemical outcome under kinetic control. This unexpected and particular stereochemical result was studied in detail by computational calculations (Figure 2). Considering that with aliphatic aldehydes the *syn*-1,2-addition products were the major diastereoisomer and showed higher enantiomeric excesses than *anti*-addition products, in order to simplify computational calculations we decided to examine in detail only the *syn*-1,2-addition pathway using organocatalyst **L_2_**. The enantiomer excess calculated with conductor-like polarizable continuum model CPCM solvation for toluene (56% ee) was in very good agreement with the experimental results (entries 2 and 3, Table 1). In our hypothesis, the presence of the polar ester group (in particular the OMe moiety) made the chain able to give electrostatic interactions with trifluoromethyl groups and with C1 (“*ex* carbonyl”), both in the *Si* face (TS-*Si-Si*, Figure 1) and in the more hindered *Re* face of the organocatalyst (*TS-Re-Re*). The same interaction, both steric and electrostatic in nature, occurred also with the carbamate group of *N*-acyliminium ion. In other words, the polar chain was a “mimic” of the *N*-acyliminium ion and *vice-versa*. Actually, the estimated ΔΔG^†^ difference calculated with CPCM, and solvation model based on density (SMD between TS-*Si-Si* and TS-*Re-R*e was only +3.2 kJ/mol (*Si-Si*/*Re-Re* = 78/22) and −0.2 kJ/mol ((*Si-Si*/*Re-Re* = 48/51), respectively, with the consequent partial loss of the high stereoinduction usually given by bulky substituent of organocatalyst L_2_ with simple aliphatic aldehydes [20].

## 3. Materials and Methods

All reagents were purchased from commercially available sources. THF and toluene were distilled on sodium/benzophenone ketyl. Solvents for extraction and chromatography were distilled before use. Analytical thin layer chromatography (TLC) were performed on silica gel on TLC Al foils (Sigma-Aldrich, St. Louis, MO, USA) with detection by exposure to ultraviolet light (254 nm) and/or by immersion in an acidic staining solution of *p*-anisaldehyde in EtOH. Merck (Kenilworth, NJ, USA) silica gel 60 (230–400 mesh) was used for flash chromatography. Semipreparative TLC were performed on Merck preparative-layer chromatography (PLC) silica gel 60. The ^1^H NMR spectra were recorded on Bruker Avance II 250 MHz spectrometer (Bruker, Billerica, MA, USA). Chemical shifts are reported in ppm downfield from tetramethylsilane with the solvent resonance as the internal standard (deuterochloroform: δ 7.26). Signal patterns are indicated as follows: br s, broad singlet; s, singlet; d, doublet; t, triplet; q, quartet; m, multiplet. Coupling constants (*J*) are given in hertz (Hz). The ^13^C NMR spectra were recorded at 62.5 MHz with complete proton decoupling. Chemical shifts are reported in ppm downfield from tetramethylsilane with the solvent resonance as the internal standard (deuterochloroform: δ 77.16). Melting points were determined with a Kofler hot-stage apparatus and are uncorrected. High resolution electron spray ionization mass spectrometry (HRESIMS) were acquired in positive ion mode with a quadrupole time-of-flights (Q-TOF) premier spectrometer (Waters-Milford, Milford, MA, USA). Analytical high-performance liquid chromatography (HPLC) was performed on a Waters 600E equipped with Varian Prostar 325 detector using a Daicel^®^ Chiralpak AD-H columns with detection at 220 nm.

### 3.1. Computational Section

Several dihedral scannings were performed pointed to individuate the most stable conformers of TS-*Si-Si* and TS-*Re-Re*. In particular, were considered several disposition of the side chain containing the ester group, the disposition of bulky substituent of organocatalyst **L_2_** (-Ar_2_OTMS group), and the disposition of the quinoline with respect to the enamine obtained by rotation around the new C-C carbon (fixed at 2.2 Å). Scanning on the potential electronic surface (PES), optimization and frequency calculation of the transition states was run with Gaussian’16 [40] (default grids and convergence criteria) at density functional theory (DFT) level PW6B95D3/def2-SVP in vacuo [41,42]. Solvent correction was estimated by single point calculation including both the CPCM [43] and SMD [44] solvation model for toluene with a larger (valence triple-zeta polarization) basis set (def2-TZVP) [42]. Subsequently, single point calculations were carried out with ORCA 4.0.1.2 [45] at spin scaled component-second order Moeller-Plesset theory with resolution-identity approximation (SCS-RIMP2/def2-TZVP) [46]. For further details see Supplementary Materials.

#### 3.1.1. General Procedure

An oven-dried 10 mL Pyrex vial was charged with 0.25 M solution (0.6 mL of THF was used for **L_1_** and toluene for **L_2_**) of the specified *N*,*O*-acetal (typically 0.15 mmol, **2a** = 35.0 mg; **2b** = 46.4 mg; **2c** = 41.3 mg; 5b = 45.8 mg). Then, 0.03 mmol of organocatalyst (**L_1_** = 7.4 mg, **L_2_** = 17.9 mg) and 0.45 mmol of aldehyde (**1a** = 52.3 mg, **1b** = 58.6 mg) were added. The resulting solution was cooled to the specified temperature and added with 0.03 mmol of In(OTf)_3_ (16.9 mg). The mixture was allowed to react until no *N,O*-acetal was detected by TLC, quenched with water (4 mL per 0.20 mmol of *N,O*-acetal), extracted four times with 5 mL of Et_2_O, and the combined organic phases were dried over MgSO_4_, filtered, and concentrated to afford a residue which was purified by chromatography or preparative TLC.

*Methyl (R)-2-((R)-5-methoxy-1,5-dioxopentan-2-yl)quinoline-1(2H)-carboxylate and methyl (R)-2-((S)-5-methoxy-1,5-dioxopentan-2-yl)quinoline-1(2H)-carboxylate (**3ab**-anti/**3ab**-syn)*

According to the general procedure, *N,O*-acetal 2a (35.0 mg, 0.15 mmol), **L_2_** (7.6 mg, 0.03 mmol), freshly distilled oxoester **1b** (58.6 mg, 0.45 mmol), In(OTf)_3_ (16.9 mg, 0.03 mmol), and toluene (0.60 mL) were allowed to react at −20 °C for 4 h. Subsequent preparative TLC (hexanes/AcOEt 8:2, 4 runs, R_f_ = 0.26) afforded an inseparable mixture of diastereoisomers as a colorless oil (48 mg, 60% yield). The ^1^H NMR (250 MHz, CDCl_3_) δ 9.59 (d, *J* = 2.2 Hz, 1H, CHO, *anti*), 9.43 (d, *J* = 3.6 Hz, 1H, CHO, *syn*), 7.55–7.37 (m, 1H, Ar-H), 7.29–7.18 (m, 1H, Ar-H), 7.15–7.05 (m, 2H, Ar-H), 6.66–6.54 (pseudo t, 1H, C*H*-CH, *anti + syn*), 6.16–6.02 (m, 1H, CH=C*H, anti + syn*), 5.41–5.30 (m, 1H, N-CH, *anti + syn*), 3.80 (s, 3H, OMe*_carb_*, *anti*), 3.78 (s, 3H, OMe*_carb_*, *syn*), 3.62 (s, 3H, OMe*_est_*, *anti*), 3.60 (s, 3H, OMe*_est_*, *syn*), and 2.62–1.74 (m, 5H, *CHCH_2_CH_2_*COOMe, *anti + syn*); ^13^C NMR (63 MHz, CDCl_3_) δ 201.8 (CHO *syn*), 201.5 (CHO *anti*), 173.3 (C=O*_est_*), 155.1 (C=O*_carb_*), 128.3 and 128.2 (C_5_, *anti + syn* )^a^, 127.2 (C_2_), 126.8 (*C*H=CH), 126.6 (CH=*C*H), 126.0 (C_3_), 125.3 (C_1_)^a^, 125.1 (C_4_)^a^, 124.9 (C_6_)^a^, 56.1 and 55.0 (*C*HCHO, *anti* + *syn*), 53.6 and 53.5 (OMe*_carb_*), 52.4 and 52.3 (N-*C*H, *anti + syn*), 51.8 (OMe*_est_*), 31.5 and 31.1 (*C*H_2_COOMe, *anti* + *syn*), and 20.6 and 20.3 (*C*H_2_CH_2_COOMe, *anti* + *syn*); ^a^tentative assignments. [M + Na]^+^ found = 340.1141, C_17_H_19_NO_5_Na^+^ requires 340.1155. The ee was determined by Daicel AD-H column (heptane–*i*-PrOH, 92:8) flow rate 1.0 mL/min; 220 nm, 3ab-*anti*: (major) = 23.1 min, *t*_R_ (minor) = 25.1 min; 32% ee; 3ab-*syn*: (minor) = 26.2 min, *t*_R_ (major) = 27.5 min; 58% ee.

*Methyl (R)-4-((R)-5-methoxy-1,5-dioxopentan-2-yl)quinoline-1(4H)-carboxylate and methyl (S)-4-((R)-5-methoxy-1,5-dioxopentan-2-yl)quinoline-1(4H)-carboxylate (**4ab**-anti/**4ab**-syn)*

Previously mentioned preparative TLC (R_f_ = 0.40) also afforded an inseparable mixture of 1,4 diastereoisomers as colorless oil (5 mg, 10%). (A = major diastereoisomer; B = minor diastereoisomer). ^1^H NMR (250 MHz, CDCl_3_) δ 9.64 (d, *J* = 2.3 Hz, 1H, CHO*_(A)_*), 9.60 (d, *J* = 2.0 Hz, 1H, CHO*_(B)_*), 8.02 (m, 1H, N-C*H*=CH, *A + B*), 7.24–7.03 (m, 4H, ArH, *A + B*), 5.34 (dd, *J* = 7.8, 5.9 Hz, 1H, N-CH=C*H_(B)_*), 5.22 (dd, *J* = 7.7, 5.7 Hz, 1H, N-CH=C*H_(A)_*), 3.95–3.82 (m, 4H, OMe*_carb_* and Ar-C*H*-CH=CH, *A + B*), 3.64 (s, 3H, OMe*_est(B)_*), 3.60 (s, 3H, OMe*_est(A)_*), 2.69–2.54 (m, 1H, C*H*CHO, *A + B*), 2.49–2.18 (m, 2H, C*H_2_*C*H_2_*COOMe, *A + B*), 2.12–1.91 (m, 2H, C*H_2_*C*H_2_*COOMe, *A + B*). ^13^C NMR (63 MHz, CDCl_3_) δ 203.1 (CHO, *A + B*), 173.3 (C=O*_est_*), 153.0 (C=O*_carb_*), 137.2 (C_2_), 128.8 (C_3_)^a^, 128.6 (C_6_)^a^, 127.6 (C_5_)^a^, 127.4 (C_4_)^a^, 125.4 (C_1_)^a^, 121.8 (*C*H=CH), 109.6 and 109.1 (CH=*C*H), 58.1 (*C*HCHO*_(B)_*), 57.9 (*C*HCHO*_(A)_*), 53.6 (OMe*_carb_*), 51.7 (OMe*_est_*), 38.6 (Ar-*C*H-CH=CH), 31.9 (*C*H_2_COOMe*_(A)_*), 31.7 (*C*H_2_COOMe*_(B)_*), 20.8 *C*H_2_CH_2_COOMe); ^a^tentative assignments. [M + Na]^+^ found = 340.1141, C_17_H_19_NO_5_Na^+^ requires 340.1155.

*Methyl (R)-2-((R)-4-methoxy-1,4-dioxobutan-2-yl)quinoline-1(2H)-carboxylate and methyl (R)-2-((S)-4-methoxy-1,4-dioxobutan-2-yl)quinoline-1(2H)-carboxylate (**3aa**-anti/**3aa**-syn)*

According to the general procedure, *N,O*-acetal **2a** (46.7 mg, 0.2 mmol), **L_1_** (10.2 mg, 0.04 mmol, 97%), oxoester **1a** (70 µL, 0.3 mmol; the commercial product had a purity of 50% because of the presence of the corresponding gemdiol), In(OTf)_3_ (22.5 mg, 0.04 mmol), THF (0.80 mL) was allowed to react at 0 °C for 4 h. Subsequent preparative TLC (petroleum ether/AcOEt 8:2, 5 runs, R_f_ = 0.38) afforded an inseparable mixture of diastereoisomers and **1a**-gemdiol as a colorless oil (30 mg, 50% yield). The ^1^H NMR (250 MHz, CDCl_3_) δ 9.78 (bs, 1H, CHO, *anti*), 9.50 (bs, 1H, CHO, *syn*), 7.56–7.38 (m, 1H, Ar-H), 7.28–7.19 (m, 1H, Ar-H), 7.16–7.07 (m, 2H, Ar-H), 6.65 (pseudo t, 1H, C*H*=CH, *anti + syn*), 6.11–5.97 (m, 1H, CH=C*H*, *anti + syn*), 5.53–5.42 (m, 1H, N-CH, *anti + syn*), 3.79 (s, 3H, OMe*_carb_*, *anti + syn*), 3.64 (s, 3H, OMe*_est_*, *syn*), 3.61 (s, 3H, OMe*_est_*, *anti*), 3.11–2.97 (m, 1H, C*H*CHO, *anti + syn*), 2.83–2.48 (m, 2H, C*H_2_*COOMe, *anti + syn*); ^13^C NMR (63 MHz, CDCl_3_) δ 200.4 (CHO, *syn*), 200.1 (CHO, *anti*), 173.6 and 172.2 (C=O*_est_*), 155.0 (C=O*_carb_*), 128.5 and 128.4 (C_5_, *anti + syn* )^a^, 127.5 (C_2_), 127.2 (*C*H=CH),), 126.7 (C_1_)^a^, 126.6 (C_4_)^a^, 126.1 (C_3_), 125.3 and 125.1 (CH=*C*H, *anti* + *syn*), 124.7 (C_6_)^a^, 53.4 (OMe*_carb_*), 52.3 and 52.1 (N-*C*H, *anti + syn*), 52.1 and 51.9 (OMe*_est_*), 51.2 (*C*HCHO, *anti* + *syn*), 29.7 and 29.5 (*C*H_2_COOMe, *anti* + *syn*); ^a^tentative assignments. [M + Na]^+^ found = 326.0987, C_16_H_17_NO_5_Na^+^ requires 326.0999. The ee was determined by Daicel OD-H column (heptane–*i*-PrOH, 85:15) flow rate 0.5 mL/min; 220 nm, **3aa**-*anti*: (major) = 12.7 min, *t*_R_ (minor) = 13.3 min; 16% ee; **3aa**-*syn*: (major) = 14.9 min, *t*_R_ (minor) = 15.8 min; 24% ee.

*Benzyl (R)-2-((R)-5-methoxy-1,5-dioxopentan-2-yl)quinoline-1(2H)-carboxylate and benzyl (R)-2-((S)-5-methoxy-1,5-dioxopentan-2-yl)quinoline-1(2H)-carboxylate (**3bb**-anti/**3bb**-syn)*

According to the general procedure, *N,O*-acetal **2b** (44.3 mg, 0.15 mmol), **L_2_** (18.5 mg, 0.03 mmol), freshly distilled oxoester **1b** (58.6 mg, 0.45 mmol), In(OTf)_3_ (16.9 mg, 0.03 mmol), toluene (0.60 mL) was allowed to react at 0 °C for 1 h. Subsequent preparative TLC (hexanes/AcOEt 8:2, 4 runs, R_f_ = 0.38) afforded an inseparable mixture of diastereoisomers as colorless oil (18 mg, 32% yield). The ^1^H NMR (250 MHz, CDCl_3_) δ 9.58 (d, *J* = 2.1 Hz, 1H, CHO, *anti*), 9.42 (d, *J* = 3.6 Hz, 1H, CHO, *syn*), 7.59–7.29 (m, 6H, Ar-H), 7.25–7.16 (m, 1H, Ar-H), 7.15–7.03 (m, 2H, Ar-H), 6.65–6.55 (pseudo t, 1H, C*H*=CH, *anti* + *syn*), 6.13–6.03 (m, 1H, CH=C*H*, *anti* + *syn*), 5.45–5.12 (m, 3H, N-CH and O-C*H_2_*-Ph, *anti* + *syn*), 3.62 (s, 3H, OMe, *syn*), 3.59 (s, 3H, OMe, *anti*), 2.65–2.52 (m, 1H, C*H*CHO, *anti + syn*), 2.43–2.10 (m, 2H, C*H_2_*C*H_2_*COOMe, *anti* + *syn*), 2.07–1.73 (m, 2H, C*H_2_*C*H_2_*COOMe, *anti*+*syn*). ^13^C NMR (63 MHz, CDCl_3_) δ 201.7 (CHO, *syn*), 201.4 (CHO, *anti*), 173.2 173.3 (C=O*_est_*), 154.4 (C=O*_carb_*), 135.9 (C_quat_)^a^, 128.7 (C_meta_)^a^, 128.6 (C_para_)^a^, 128.4 (C_5_)^a^, 128.2 (C_orto_)^a^, 127.2 (C_2_), 126.9 (*C*H=CH), 126.7 (CH=*C*H), 125.3 (C_1_)^a^, 125.1 (C_4_)^a^, 125.0 (C_6_)^a^, 121.9 (C_3_), 68.4 (O*C*H_2_Ph), 56.2 (N-*C*H, *anti + syn*), 52.4 (OMe*_est_*), 51.8 *C*HCHO, *anti* + *syn*), 31.5 and 31.2 (*C*H_2_COOMe, *anti* + *syn*), 20.6 and 20.4 (*C*H_2_CH_2_COOMe, *anti* + *syn*); ^a^tentative assignments. [M + Na]^+^ found = 416.1451, C_19_H_25_NO_5_Na^+^ requires 416.1468.

*Benzyl (R)-2-((R)-4-methoxy-1,4-dioxobutan-2-yl)quinoline-1(2H)-carboxylate and benzyl (R)-2-((S)-4-methoxy-1,4-dioxobutan-2-yl)quinoline-1(2H)-carboxylate (**3ba**-anti/**3ba**-syn).*

According to the general procedure, *N,O*-acetal **2b** (31.7 mg, 0.11 mmol), **L_2_** (13.6 mg, 0.022 mmol), oxoester **1a** (38 mg, 0.33 mmol, 50%; because of presence of corresponding gemdiol), In(OTf)_3_ (12.4 mg, 0.022 mmol), toluene (0.55 mL) was allowed to react at 0 °C for 1 h. Subsequent preparative TLC (petroleum ether/AcOEt, 4 runs, R_f_ = 0.34) afforded an inseparable mixture of diastereoisomers as colorless oil (18 mg, 45% yield). The ^1^H NMR (250 MHz, CDCl_3_) δ 9.75 (s, 1H, CHO, *anti*), 9.50 (s, 1H, CHO, *syn*), 7.55–7.28 (m, 6H, Ar-H), 7.26–7.16 (m, 1H, Ar-H), 7.16–7.05 (m, 2H, Ar-H), 6.68–6.58 (pseudo t, 1H, C*H*=CH, *anti* + *syn*), 6.10–5.99 (m, 1H, CH=C*H*, *anti* + *syn*), 5.55–5.45 (m, 1H, N-CH, *anti* + *syn*), 5.37–5.12 (m, 2H, O-CH_2_-Ph, *anti* + *syn*), 3.60 (s, 3H, OMe, *syn*), 3.57 (s, 3H, OMe, *anti*), 3.11–2.98 (m, 1H, C*H*CHO, *anti + syn*), 2.81–2.54 (m, 1H, C*H_2_*COOMe, *anti* + *syn*), 2.52–2.27 (m, 1H, C*H_2_*COOMe, *anti* + *syn*). The ^13^C NMR (63 MHz, CDCl_3_) δ 200.3 (CHO, *syn*), 200.1 (CHO, *anti*), 172.1 (C=O*_est_*), 154.4 (C=O*_carb_*), 135.7 (C_quat_)^a^, 128.7 (C_meta_)^a^, 128.5 (C_para_)^a^, 128.4 (C_5_)^a^, 128.3 (C_orto_)^a^, 128.2 (C_orto_)^a^, 127.6 (C_2_), 127.2 (*C*H=CH, *anti + syn*), 126.7 (C_1_)^a^, 126.7 (C_4_)^a^, 126.1 (C_3_), 125.3 and 125.2 (CH=*C*H, *anti + syn*), 124.8 (C_6_)^a^, 68.5 and 68.4 (O*C*H_2_Ph, *anti* + *syn*), 53.4 (N-*C*H, *anti + syn*), 52.1 and 52.0 (OMe*_est_*), 51.2 (*C*HCHO), 29.7 and 29.5 (*C*H_2_COOMe, *anti* + *syn*); ^a^tentative assignments. [M + Na]^+^ found = 402.1301, C_19_H_25_NO_5_Na^+^ requires 402.1312.

*Isobutyl (R)-2-((R)-5-methoxy-1,5-dioxopentan-2-yl)quinoline-1(2H)-carboxylate and isobutyl (R)-2-((S)-5-methoxy-1,5-dioxopentan-2-yl)quinoline-1(2H)-carboxylate **(3cb**-anti/**3cb**-syn) and isobutyl (S)-4-((R)-5-methoxy-1,5-dioxopentan-2-yl)quinoline-1(4H)-carboxylate and isobutyl (S)-4-((S)-5-methoxy-1,5-dioxopentan-2-yl)quinoline-1(4H)-carboxylate (**4cb**-anti/**4cb**-syn)*

According to the general procedure, *N,O*-acetal **2c** (45.5 mg, 0.15 mmol), **L_2_** (18.5 mg, 0.03 mmol, 97%), freshly distilled oxoester **9b** (55 µL, 0.45 mmol), In(OTf)_3_ (16.9 mg, 0.03 mmol), toluene (0.60 mL) was allowed to react at 0 °C for 2 h. Subsequent preparative TLC (petroleum ether/AcOEt 8:2, 3 runs, R_f_ = 0.49) afforded an inseparable mixture of regioisomers as colorless oil (36 mg, 66% yield). The ^1^H NMR (250 MHz, CDCl_3_) δ 9.78–9.75 (m, 1H, CHO*_1,4-A_*), 9.63 (d, *J* = 2.2 Hz, 1H, CHO*_1,4-B_*), 9.59 (d, *J* = 2.2 Hz, 1H, CHO*_1,2-anti_*), 9.44 (d, *J* = 3.6 Hz, 1H, CHO*_1,2-syn_*), 8.05–7.98 (m, 1H, N-C*H*=CH*_1,4-A+B_*), 7.60–7.36 (m, 1H, Ar-H*_1,2/1,4_*), 7.29–7.14 (m, 1H, Ar-H*_1,2/1,4_*), 7.19–7.03 (m, 2H, Ar-H*_1,2/1,4_*and 1H, N-CH=C*H_1,4-A+B_*), 6.59 (pseudo t, 1H, C*H*=CH*_1,2-anti+syn_*), 6.17–6.00 (m, 1H, CH=C*H_1,2-anti+syn_*), 5.35 (pseudo t, 1H, N-CH*_1,2-anti+syn_*), 5.21 (m, 1H, N-CH=CH-C*H_1,4-A+B_*), 4.12–3.84 (m, 2H, (CH_3_)_2_CHC*H_2_*O*_1,2/1,4_*), 3.67 (s, 3H, OMe*_1,4-A+B_*), 3.62 (s, 3H, OMe*_1,2-anti_*), 3.59 (s, 3H, OMe*_1,2-syn_*), 2.63–2.49 (m, 1H, C*H*CHO*_1,2/1,4_*), 2.44–2.17 (m, 2H, C*H_2_*C*H_2_*COOMe*_1,2/1,4_*), 2.11–1.77 (m, 2H C*H_2_*C*H_2_*COOMe*_1,2/1,4_*),0.99 (d, *J* = 6.7 Hz, 6H, 2 x (CH_3_) *_1,4-A+B_*), 0.94 (d, *J* = 4.0 Hz, 6H, 2 x (CH_3_)*_1,2-anti_*), 0.91 (d, *J* = 4.0 Hz, 6H, 2 x (CH_3_)*_1,2-syn_*). The ^13^C NMR (63 MHz, CDCl_3_) δ 201.8 (CHO*_syn_*), 201.6 (CHO*_anti_*), 173.3 (*C*OOMe*_syn_*), 173.1 (*C*OOMe*_anti_*), 154.7 (*C*OOMe*_anti_*), 154.6 (*C*OOMe*_syn_*), 134.5 and 134.3 (C_2_), 128.1 and 128.0 (C_5_)^a^, 127.2 (*C*H=CH), 126.8 (C_1_)^a^, 126.6 (C_4_)^a^, 126.5 (CH=*C*H), 125.4 (C_3_), 125.1 and 125.0 (C_6_)^a^, 72.9 (*C*H_2_OOC-N, *anti + syn*), 56.1 (*C*HCHO*_syn_*), 55.0 (*C*HCHO*_anti_*), 52.3 (N-CH*_syn_*), 52.2 (N-CH*_anti_*), 51.8 (OMe*_anti_*), 51.7 (OMe*_syn_*), 31.5 (*C*H_2_COOMe*_anti_*), 31.2(*C*H_2_COOMe*_syn_*), 27.9 ((CH_3_)_2_CH, *anti + syn*), 20.6 (*C*H_2_CH_2_COOMe*_anti_*), 20.4 (*C*H_2_CH_2_COOMe*_syn_*), 19.3 ((CH_3_)*_anti_*), 19.2 ((CH_3_)*_anti_*); ^a^tentative assignments.

*Tert-Butyl-(R)-1-((S)-5-methoxy-1,5-dioxopentan-2-yl)-3,4-dihydroisoquinoline-2(1H)-carboxylate/tert-butyl-(R)-1-((R)-5-methoxy-1,5-dioxopentan-2-yl)-3,4-dihydroisoquinoline-2(1H)-carboxylate (**6b**-anti/**6b**-syn).*

According to the general procedure, *N,O*-acetal **5b** (47.8 mg, 0.157 mmol), **L_1_** (8.0 mg, 0.031 mmol), freshly distilled oxoester **1b** (61.2 mg, 0.47 mmol), In(OTf)_3_ (17.6 mg, 0.031 mmol), THF (0.60 mL) was allowed to react at 0 °C for 3 h. Subsequent preparative TLC (petroleum ether/AcOEt 8:2, 2 runs, R_f_ = 0.39) afforded an inseparable mixture of diastereoisomers as a colorless oil (36 mg, 60% yield). (A = major diastereoisomer; B = minor diastereoisomer). The ^1^H NMR (250 MHz, CD_3_CN, 65°C) δ 9.72–9.67 (bs, 1H, CHO*_(B)_*), 9.63–9.58 (bs, 1H, CHO*_(A)_*), 7.30–7.11 (m, 4H, Ar-H, *A + B*), 5.55–5.47 (m, 1H, Ar-*CH-*N_(*B)*_), 5.40–5.31 (m, 1H, Ar-*CH-*N_(*A)*_), 3.86–3.67 (m, 1H, 1 x N-C*H_2_, (A + B)*), 3.58 (s, 3H, OMe_(*A)*_), 3.57 (s, 3H, OMe_(*B)*_), 3.41–3.26 (m, 1H, 1 x N-C*H_2_, (A + B)*), 2.95–2.81 (m, 2H, Ar-C*H_2_, (A + B)*), 2.80–2.65 (m, 1H, C*H*CHO, *(A + B)*), 2.38–2.12 (m, 3H, *CH_2_CH_2_*COOMe, *(A + B)*), 1.81–1.62 (m, 1H, *CH_2_CH_2_*COOMe, *(A + B)*), 1.45 (s, 9H, C(C*H_3_*)_3*(A)*_), 1.44 (s, 9H, C(C*H_3_*)_3*(B)*_); ^13^C NMR (63 MHz, CDCl_3_) δ 203.0 (CHO*_(A)_*), 202.1 (CHO*_(B)_*), 173.5 (*C*OOMe*_(A)_*), 173.2 (*C*OOMe*_(B)_*), 155.5 (*C*OO*t*-Bu*_(A)_*), 150.8 (*C*OO*t*-Bu*_(B)_*), 135.1 (C_2_), 134.8 (C_3_)^a^, 129.1 (C_4_, *B*)^a^, 128.8 (C_4_, *B*)^a^, 127.8 (C_5_)^a^, 127.4 (C_6_)^a^, 126.7 (C_1_, *B*)^a^, 126.5 (C_1_, *B*)^a^, 81.1 (*C*(CH_3_)_3*(B)*_), 80.5 (*C*(CH_3_)_3*(A)*_), 58.7 (Ar-C*H_2_, A + B*), 58.5 (*C*HCHO, *A+B*), 55.1 (Ar-*CH-*N_(A*)*_), 53.6 (Ar-*CH-*N_(*B)*_), 51.8 (OMe, *A+B*), 40.9 and 40.4* (*C*H_2_*-*N_(*A)*_), 39.4 and 38.9* (*C*H2*-*N_(*B)*_), 31.8 (*C*H_2_COOMe_(*B)*_), 31.6 (*C*H_2_COOMe_(*A)*_), 28.5 (CH_3*(A)*_), 28.0 (CH_3*(B)*_), 22.0 (*CH_2_CH_2_*COOMe*_(A)_*), 20.7 (*CH_2_CH_2_*COOMe*_(B)_*); ^a^tentative assignments (*minor rotamer). [M + Na]^+^ found = 384.1772, C_20_H_27_NO_5_Na^+^ requires 384.1781. The ee was determined only for the major diastereoisomer by Daicel AD-H column (heptane–*i*-PrOH, 92:8) flow rate 1.0 mL/min; 220 nm, (minor) = 11.9 min, *t*_R_ (major) = 12.8 min; 70% ee.

*Tert-Butyl-(R)-1-((S)-4-methoxy-1,4-dioxobutan-2-yl)-3,4-dihydroisoquinoline-2(1H)-carboxylate and tert-butyl (R)-1-((R)-4-methoxy-1,4-dioxobutan-2-yl)-3,4-dihydroisoquinoline-2(1H)-carboxylate (**6a**-anti/**6a**-syn).*

According to the general procedure, *N,O*-acetal **5b** (45.8 mg, 0.15 mmol), **L_1_** (7.6 mg, 0.03 mmol, 97%), oxoester **1a** (52.4 µL, 0.225 mmol, 50% purity because of presence of corresponding gemdiol), In(OTf)_3_ (16.9 mg, 0.03 mmol), THF (0.60 mL) was allowed to react at 0 °C for 2 h. Subsequent preparative TLC (petroleum ether/AcOEt 8:2, 3 runs, R_f_ = 0.49) afforded an inseparable mixture diastereoisomers as a colorless oil (27 mg, 52% yield). (A = major diastereoisomer; B = minor diastereoisomer). The ^1^H NMR (250 MHz, CD_3_CN, 65°C) δ 9.77 (d, *J* = 1.4 Hz, 1H, CHO*_(A)_*), 9.68 (d, *J* = 1.8 Hz, 1H, CHO*_(B)_*), 7.31–7.14 (m, 4H, Ar-H, *A + B*), 5.57 (d, *J* = 6.6 Hz, 1H, Ar-*CH*-N*_(A)_*), 5.44 (d, *J* = 7.6 Hz, 1H, Ar-*CH*-N*_(B)_*), 3.99–3.73 (m, 1H, 1 x *CH_2_*N, *A + B*), 3.58 (s, 3H, OMe, *A+B*), 3.46–3.23 (m, 2H, 1 x *CH_2_* and *CH*CHO, *A+B*), 2.94–2.66 (m, 3H, 1 x Ar-*CH_2_* and *CH_2_*COOMe, *A + B*), 2.41 (m, 1H, 1 x Ar-*CH_2_*, *A + B*), 1.46 (s, 9H, C(*CH_3_*)_3_, *A + B*); ^13^C NMR (63 MHz, CD_3_CN) δ 202.7 (CHO, *A+B*), 173.1 (*C*OOMe*_(A)_*), 173.0 (*C*OOMe*_(B)_*), 153.7 (*C*OO*t*-Bu, *A + B*), 136.6 (C_2_), 129.9 (C_3_)^a^, 128.6 (C_4_)^a^, 128.3 (C_5_)^a^, 128.0 (C_6_)^a^, 127.4 (C_1_, *B*)^a^, 127.2 (C_1_, *A*)^a^, 81.2 (*C*(CH_3_)_3*(B)*_), 80.9 (*C*(CH_3_)_3*(A)*_), 56.3 (*C*HCHO*_(A)_*), 54.6 (*C*HCHO*_(B)_*), 54.6 (Ar-*CH-*N_(A*)*_), 54.2 (Ar-*CH-*N_(*B)*_), 52.3 (OMe*_(A)_*), 52.3 (OMe*_(B)_*), 41.5 (*C*H_2_*-*N_(*A)*_), 40.2 (*C*H_2_*-*N_(*B)*_), 31.7 (Ar-*C*H*_2(A)_*), 30.8 (Ar-*C*H*_2(B)_*), 28.8 *C*H_2_COOMe, *A + B*), 28.5 (CH_3_, *A + B*); ^a^tentative assignments. [M + Na]^+^ found = 370.1608, C_19_H_25_NO_5_Na^+^ requires 370.1625. The ee was determined by Daicel AD-H column (heptane–*i*-PrOH, 92:8) flow rate 1.0 mL/min; 220 nm, **6a**-*anti*: (major) = 21.7 min, *t*_R_ (minor) = 28.8 min; 68% ee; **6a**-*syn*: (minor) = 15.7 min, *t*_R_ (major) = 25.8 min; 2% ee.

#### 3.1.2. General Procedure for Lactonization

A round-bottomed flask was charged with 0.15 mmol of oxoester (1.0 eq **3aa** = 45.5 mg, **3ab** = 47.6 mg, **6a** = 45.8 mg) and MeOH (0.7 mL). The solution was cooled at 0 °C, and 0.6 mmol of NaBH_4_ (22.7 mg) was added portion-wise. The reaction was allowed to react until no starting material was detected with TLC. The mixture was quenched with H_2_O (4 mL per 0.2 mmol of oxoester) and the aqueous layer was extracted four times with Et_2_O (5 mL). The combined organic layers were dried over MgSO_4_, filtered, and concentrated to afford a residue, which was purified by flash chromatography or/and preparative TLC.

*Methyl 2-(6-oxotetrahydro-2H-pyran-3-yl)quinoline-1(2H)-carboxylate (**7b**)*

According to the general procedure, a 10-mL round-bottomed flask was loaded with **3ab** (50.0 mg, 0.16 mmol), MeOH (0.75 mL). and NaBH_4_ (24.7 mg, 0.64 mmol). After 30 min, the standard workup provided a crude which was purified with preparative TLC (petroleum ether/AcOEt 6:4, 3 runs, R_f_ = 0.48), affording **7b** as an amorphous white solid (25 mg, 54% yield). (A = major diastereoisomer; B = minor diastereoisomer). The ^1^H NMR (250 MHz, CD_3_Cl) δ 7.53–7.03 (m, 4H, Ar-H), 6.60 (m, 1H, *CH*=CH, *A+B*), 6.11–5.99 (m, 1H, CH=*CH*, *A + B*), 5.26–5.14 (m, 1H, N-*CH_(B)_*), 5.07–4.89 (m, 1H, N-*CH_(A)_*), 4.36–4.05 (m, 1H, *CH_2_*O-C=O*_(A)_*), 3.80 (s, 3H, OMe*_(A)_*), 3.58 (s, 3H, OMe*_(A)_*), 3.51–3.40 (m, 1H, *CH_2_*O-C=O*_(B)_*), 2.73–2.54 (m, 1H, 1 x *CH_2_*-C=O, *A + B*), 2.49–2.31 (m, 1H, 1 x *CH_2_*-C=O, *A + B*), 2.13–1.66 (m, 3H, *CH*C*H_2_*CH_2_C=O, *A + B*); ^13^C NMR (63 MHz, CDCl_3_) δ 171.2 (C=O*_est(A)_*), 171.1 (C=O*_est(B)_*), 155.3 (C=O*_carb(A)_*), 155.1 (C=O*_carb(B)_*), 134.9 (C_2_, *B*)^a^, 134.4 and 134.2 (C_2_, *A*)^a^, 128.8 (CH=*C*H*_(B)_*), 128.2 (C_5_, *A*)^a^, 127.7 (C_5_, *B*)^a^, 127.2 (C_3_), 126.9 (C_1_, *A*)^a^, 126.8 (C_1_, *B*)^a^, 126.7 (*C*H=CH*_(A)_*), 126.4 (CH=*C*H*_(A)_*), 126.0 (CH=*C*H*_(B)_*), 125.2 (C_4_, *A*)^a^, 125.1 (C_4_, *B*)^a^, 125.0 (C_6_)^a^, 70.3* and 70.0 (*C*H_2_O-C=O*_(A)_*), 60.9 and 60.5* (*C*H_2_O-C=O*_(B)_*), 53.5 (N-*C*H*_(B)_*), 53.3 (OMe*_(A)_*), 52.7 (N-*C*H*_(A)_*), 51.7 (OMe*_(B)_*), 45.2 (*C*HCH_2_O-C=O*_(B)_*), 37.0* and 36.5 (*C*HCH_2_O-C=O*_(A)_*), 28.8 (*C*H_2_-C=O*_(B)_*), 28.7 (*C*H_2_-C=O*_(A)_*), 21.8 (*C*H_2_CH_2_C=O*_(B)_*), 21.6 (*C*H_2_CH_2_C=O*_(A)_*); ^a^tentative assignments (*minor rotamer). [M + Na]^+^ found = 310.1041, C_16_H_17_NO_4_Na^+^ requires 310.1050.

*Methyl 2-(5-oxotetrahydrofuran-3-yl)quinoline-1(2H)-carboxylate (**7a**)*

According to the general procedure, a 10-mL round-bottomed flask was loaded with **3aa** (36.4 mg, 0.105 mmol), MeOH (0.50 mL), and NaBH_4_ (16.2 mg, 0.42 mmol). After 45 min, the standard workup provided a crude which was purified with preparative TLC (petroleum ether/AcOEt 6:4, 2 runs, R_f_ = 0.23), affording **7a** as a colorless oil (15 mg, 52% yield). (A = major diastereoisomer; B = minor diastereoisomer). The ^1^H NMR (250 MHz, CDCl_3_) δ 7.56–7.39 (m, 1H, Ar-H), 7.31–7.19 (m, 1H, Ar-H), 7.17–7.08 (m, 2H, Ar-H), 6.62 (d, *J* = 9.6 Hz, 1H, *CH*=CH, *A+B*), 6.01 (dd, *J* = 9.6, 5.8 Hz, 1H, CH=*CH*, *A+B*), 5.14–5.02 (m, 1H, N-*CH*, *A+B*), 4.32–4.19 (m, 1H, 1 x *CH_2_*O-C=O, *A+B*), 4.18–4.07 (m, 1H, 1 x *CH_2_*O-C=O, *A + B*), 3.81 (s, 3H, OMe, *A + B*), 2.77–2.59 (m, 1H, *CH*CH_2_O-C=O, *A + B*), 2.47–2.36 (m, 2H, *CH_2_*-C=O, *A + B*); ^13^C NMR (63 MHz, CDCl_3_) δ 176.3 (C=O*_est(B)_*), 176.1 (C=O*_est(A)_*), 155.3 (C=O*_carb_*, *A + B*), 134.3 and 134.3 (C_2_), 128.4 and128.3 (C_5_)^a^, 127.5 and 127.4 (*C*H=CH, *A + B*), 127.0 (C_3_), 126.7 (C_1_)^a^, 125.8 and 125.5 (CH=*C*H, *A + B*), 125.2 (C_1_)^a^, 125.0 and 124.9 (C_6_)^a^, 70.1 (*C*H_2_O-C=O*_(B)_*), 69.4 (*C*H_2_O-C=O*_(A)_*), 53.8 (N-*C*H*_(B)_*), 53.6 (OMe, *A + B*), 53.5 (N-*C*H*_(B)_*), 39.9 (*C*HCH_2_O-C=O*_(A)_*), 39.1 (*C*HCH_2_O-C=O*_(B)_*), 31.0 (*C*H_2_-C=O*_(A)_*), 30.9 (*C*H_2_-C=O*_(A)_*); ^a^tentative assignments. [M + Na]^+^ found = 296.0880, C_15_H_15_NO_4_Na^+^ requires 296.0893.

*Tert-Butyl 1-(5-oxotetrahydrofuran-3-yl)-3,4-dihydroisoquinoline-2(1H)-carboxylate (**8**)*

According to the general procedure, a 10-mL round-bottomed flask was loaded with **6a** (88.0 mg, 0.253 mmol), MeOH (1.2 mL), and NaBH_4_ (38.6 mg, 1.0 mmol). After 1 h, the standard workup provided a crude which was purified with preparative TLC (petroleum ether:AcOEt 7:3, 2 runs, R_f_ = 0.58), affording **8** as an amorphous white solid (36 mg, 45% yield). (A = major diastereoisomer; B = minor diastereoisomer). The ^1^H NMR (250 MHz, CDCl_3_) δ 7.27–6.97 (m, 4H, Ar-H), 5.24 (d, *J* = 9.0 Hz, 1H, Ar-C*H*-N*_(A)_*), 4.97 (dd, *J* = 9.6, 4.0 Hz, 1H, Ar-C*H*-N*_(A)_*), 4.37–4.13 (m, 2H, *C*H_2_O-C=O, *A + B*), 4.10–3.92 (m, 1H, 1 x *CH_2_*-C=O*_(B)_*), 3.90–3.69 (m, 1H, 1 x *CH_2_*-C=O*_(A)_*), 3.53–3.29 (m, 1H, 1 x *CH_2_*-C=O, *A + B*), 3.07–2.75 (m, 3H, C*H*CH_2_O-C=O and Ar-C*H_2_*, *A + B*), 2.66–2.38 (m, 2H, C*H_2_*-N, *A + B*), 1.49 (s, 9H, C(*CH_3_*)_3*(B)*_), 1.47 (s, 9H, C(*CH_3_*)_3*(A)*_). The ^13^C NMR (63 MHz, CDCl_3_) δ 176.6 (C=O*_est_*, *A + B*), 155.7 (*C*OO*t*-Bu, *A + B*), 135.3 (C_2_), 134.8 (C_3_, *B*)^a^, 134.7 (C_3_, *A*)^a^, 129.5 (C_4_, *B*)^a^, 129.1 (C_4_, *A*)^a^, 128.0 and 127.9 (C_5_, *B*)^a^, 127.8 and 127.8 (C_5_, *A*)^a^, 127.0 and 126.9 (C_6_, *B*)^a^, 126.8 and 126.7 (C_6_, *A*)^a^, 126.6 (C_1_, *A*)^a^,126.6 (C_1_, *A*)^a^, 80.6 (*C*(CH_3_)_3*(B)*_), 80.6 (*C*(CH_3_)_3*(A)*_), 71.0 and 70.8* (*C*H_2_O-C=O*_(A)_*), 70.8 and 70.6 (*C*H_2_O-C=O*_(B)_*), 56.7* and 56.4 (Ar-C*H*-N*_(B)_*), 55.8* and 55.6 (Ar-C*H*-N*_(B)_*), 43.0 and 42.5* (*C*HCH_2_O-C=O*_(A)_*) 42.4 and 42.2* (*C*HCH_2_O-C=O*_(B)_*), 40.5 and 40.2* (*C*H_2_-C=O*_(A)_*), 39.1 and 39.0* (*C*H_2_-C=O*_(B)_*), 33.1* and 33.0 (*C*H_2_-N*_(B)_*), 32.5* and 32.3 (*C*H_2_-N*_(B)_*), 28.5 and 28.3* (CH_3_), 27.8 (Ar-*C*H_2_); ^a^tentative assignments (*minor rotamer). [M + Na]^+^ found = 340.1505, C_18_H_23_NO_4_Na^+^ requires 340.1519. The ee was determined by Daicel AD-H column (heptane–*i*-PrOH, 90:10) flow rate 1.0 mL/min, 220 nm, major diasteroisomer: (minor) = 16.5 min, *t*_R_ (major) = 18.1 min; 43% ee; minor diasteroisomer: (minor) = 31.5 min, *t*_R_ (major) = 33.6 min; 35% ee.

## 4. Conclusions

A novel catalytic enantioselective direct α-amidoalkylation of quinolines and isoquinolines with γ- and δ-oxoesters was studied in detail. The synergistic combination of a Lewis acid and chiral secondary amine organocatalysts allowed in situ-generation of the reactive species in an acyl Mannich-type reaction. The low to moderate facial selectivity obtained with these particular enolizable substrates was rationalized computationally, pointing to steric and electrostatic interactions of the remote ester group with the bulky substituents of the organocatalyst and with the former aldehyde carbon. The newly obtained polyfunctionalized products included new heterofunctionalized γ- and δ-lactones.

## Figures and Tables

**Table 1:** Results of N-acyl Mannich reaction of oxoesters **1a,b** with *N,O*-acetals **2a**–**c** *^a^*.

Entry	Sub	L	Oxoester	T (h)	1,2/1,4 *^b^* (3/4)	Dr *^b^* *(Syn/Anti)*	Ee *(Syn/Anti)*	Yield (%) *^c^*
1	**2a**	**L1**	**1b**	1	83/17	42/58	10/32	26(**3ab**)
2	**2a**	**L2**	**1b**	1	89/11	60/40	43/10	44(**3ab**)
3 *^d^*	**2a**	**L2**	**1b**	4	89/11	60/40	58/32	60(**3ab**)
4 *^d^*	**2a**	**L1**	**1b**	4	95/5	50/50	5/12	30(**3ab**)
5	**2a**	**L1**	**1a**	4	86/14	57/43	16/24	50(**3aa**)
6	**2a**	**L2**	**1a**	3	88/12	60/40	28/20	84(**3aa**)
7	**2b**	**L1**	**1b**	1	85/15	50/50	nd	14(**3bb**)
8	**2b**	**L2**	**1b**	1	89/11	70/30	nd	32(**3bb**)
9	**2b**	**L1**	**1a**	1	80/20	56/44	nd	25(**3ba**)
10	**2b**	**L2**	**1a**	1	83/17	68/32	nd	33(**3ba**)
11	**2c**	**L2**	**1a**	2	80/20	62/38	nd	66(**3cb**) *^f^*

*^a^* All reactions were carried out in accordance with the general procedure. *^b^* Determined by ^1^H NMR of the crude mixture. *^c^* Isolated yield of the indicated products after chromatographic purification. *^d^* Reaction carried out at −20 °C. *^f^* Regioisomeric 1,2 and 1,4-adducts were inseparable. Nd: Not determined.

**Table 2:** Results of *N*-acyl Mannich reaction of oxoesters **1a,b** with *N,O*-acetals **5a,b***^a^*.

Entry	Sub	L	Oxoester	T (h)	Dr *^b^*	ee	Yield (%) *^c^* (Product)
1	**5a**	**L1/L2**	**1a**	3	Na	Low conversion	
2	**5b**	**L1**	**1b**	3	65/35	70/nd	60 (**6b**)
3	**5b**	**L2**	**1b**	3	50/50	50/nd	32 (**6b**)
4	**5b**	**L1**	**1a**	2	68/32	70/2	52 (**6a**)
5	**5b**	**L2**	**1a**	15	45/55	68/30	34 (**6a**)

*^a^* All reactions were carried out in accordance with the general procedure. *^b^* Determined by ^1^H NMR of the crude mixture. *^c^* Isolated yield of the indicated products after chromatographic purification. Nd: Not determined.

## Supplementary Materials

Copies of ^1^H and ^13^C NMR of all new products and computational details are provided as Supplementary Materials available online.
