# Supplementary material for: Experimental and Computational Studies Unraveling the Peculiarity of Enolizable Oxoesters in the Organocatalyzed Mannich-Type Addition to Cyclic N-Acyl Iminium Ions
Source: Molecules. 2020 Apr 20;25(8):1903. doi: 10.3390/molecules25081903 (PMC7221655; doi:10.3390/molecules25081903)
Supplement: Supplementary file 1 [file molecules-25-01903-s001.pdf]

# Supporting Information for

# Experimental and theoretical studies unraveling the peculiarity of enolizable oxoesters in the organocatalyzed Mannich-type addition to cyclic N-acyl iminium ions

Andrea Menichetti,[a] Sebastiano Di Pietro,[a] Valeria Di Bussolo,[b] Lucilla Favero,[a]* and Mauro Pineschi[a]*

[a]*Department of Pharmacy, University of Pisa, via Bonanno 33, 56126, Pisa, Italy*

[b]*Department of Chemistry and Industrial Chemistry, University of Pisa, via Moruzzi 3, 56124, Pisa, Italy.*[1]

*Email: mauro.pineschi@farm.unipi.it*

**Contents**

$^1$H and $^{13}$C NMR spectra of products . . . . . . . . . . . . . . . . . . . . . . . . . . . . . . . . . . . . . . . . . . . . . . . . . .S1

Computational data . . . . . . . . . . . . . . . . . . . . . . . . . . . . . . . . . . . . . . . . . . . . . . . . . . . . . . . . . . . . . .S23

Compound **3ab-*anti*/3ab-*syn*** $^{1}$H NMR (250 MHz, $CDCl_3$)

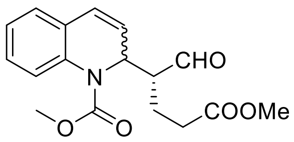


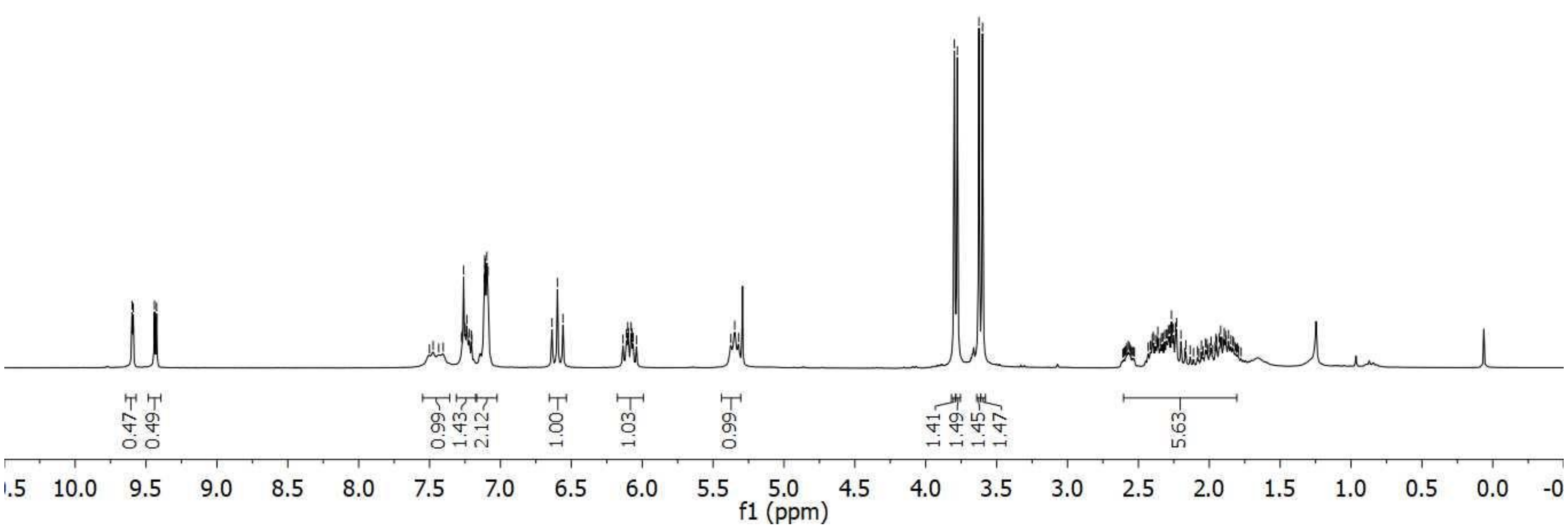

Compound **3ab-*anti*/3ab-*syn*** $^{13}$C NMR (62.5 MHz, $CDCl_3$)

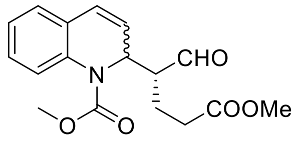


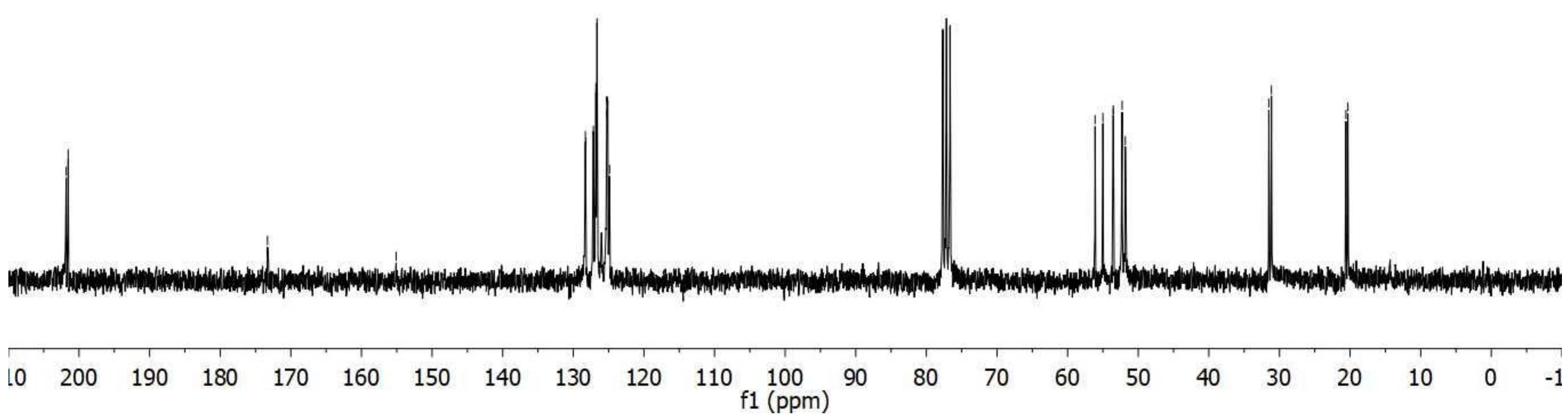

Compound **4ab-*anti*/4ab-*syn*** $^{1}$H NMR (250 MHz, $CD_3Cl$)

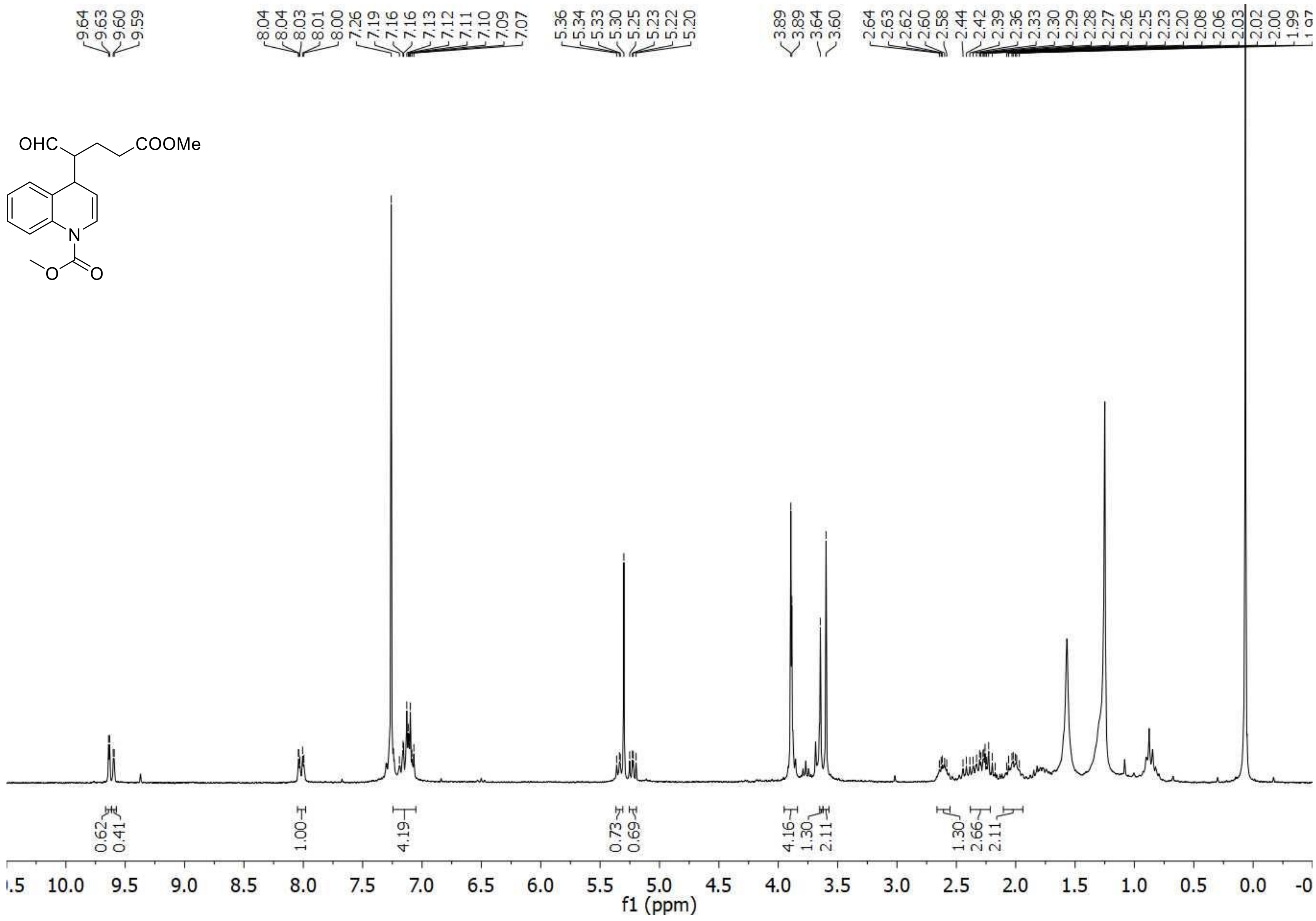

Compound **4ab-*anti*/4ab-*syn*** $^{13}$C NMR (62.5 MHz, $CD_3Cl$)

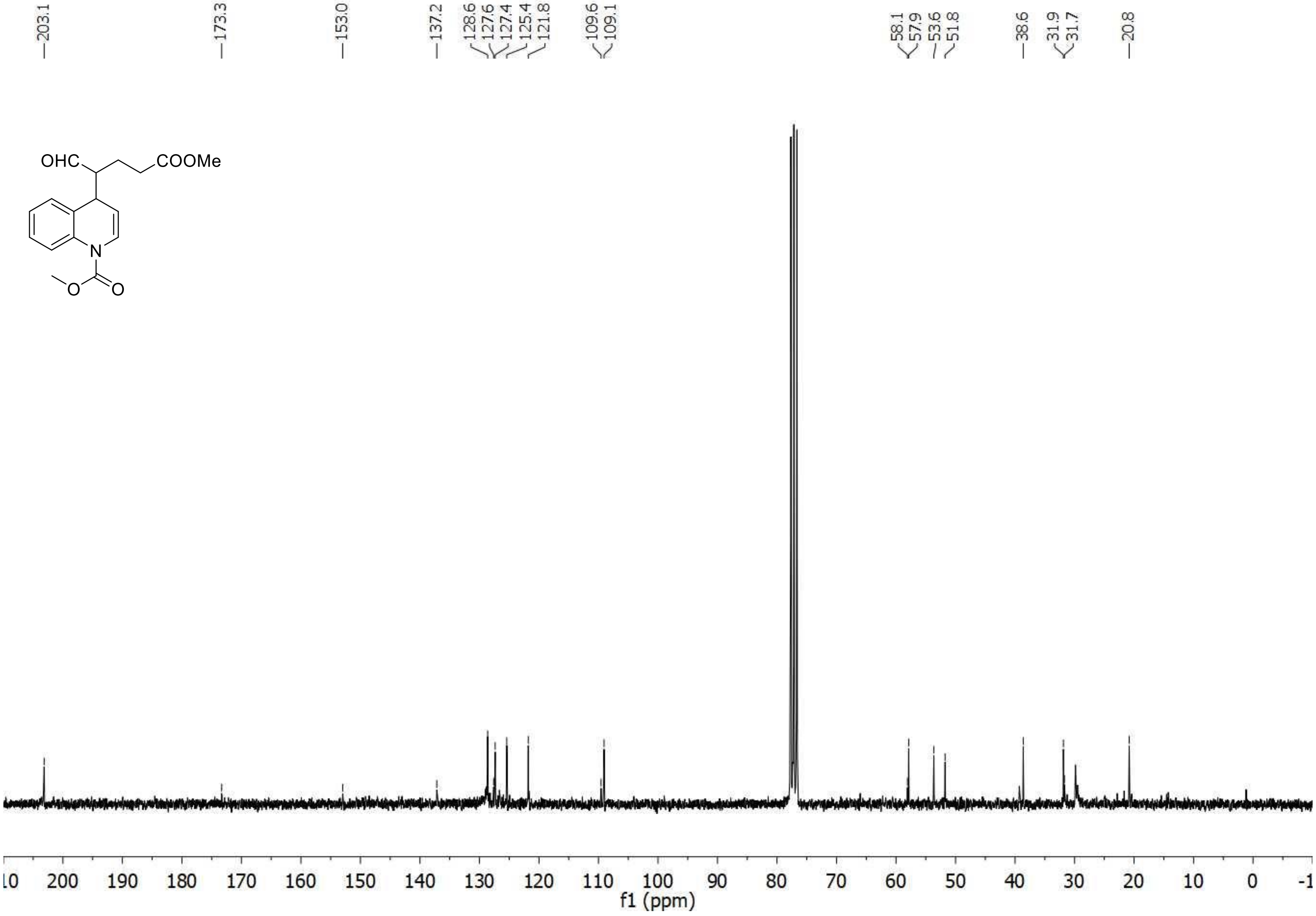

Compound 3**aa-*anti***/**3aa-*syn*** $^{1}$H NMR (250 MHz, $CD_3Cl$)

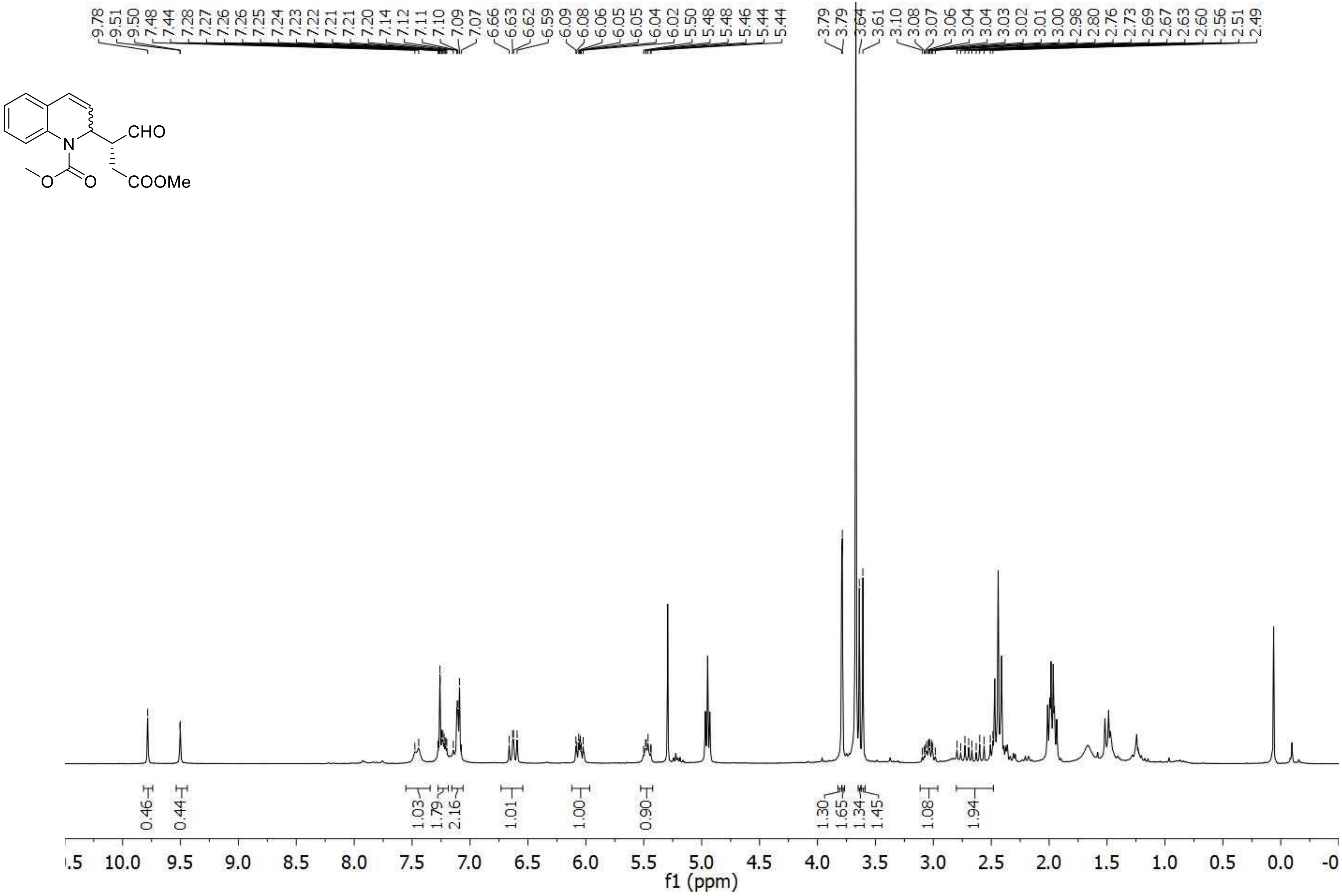

Compound **3aa-*anti*/3aa-*syn*** $^{13}$C NMR (62.5 MHz, $CDCl_3$)

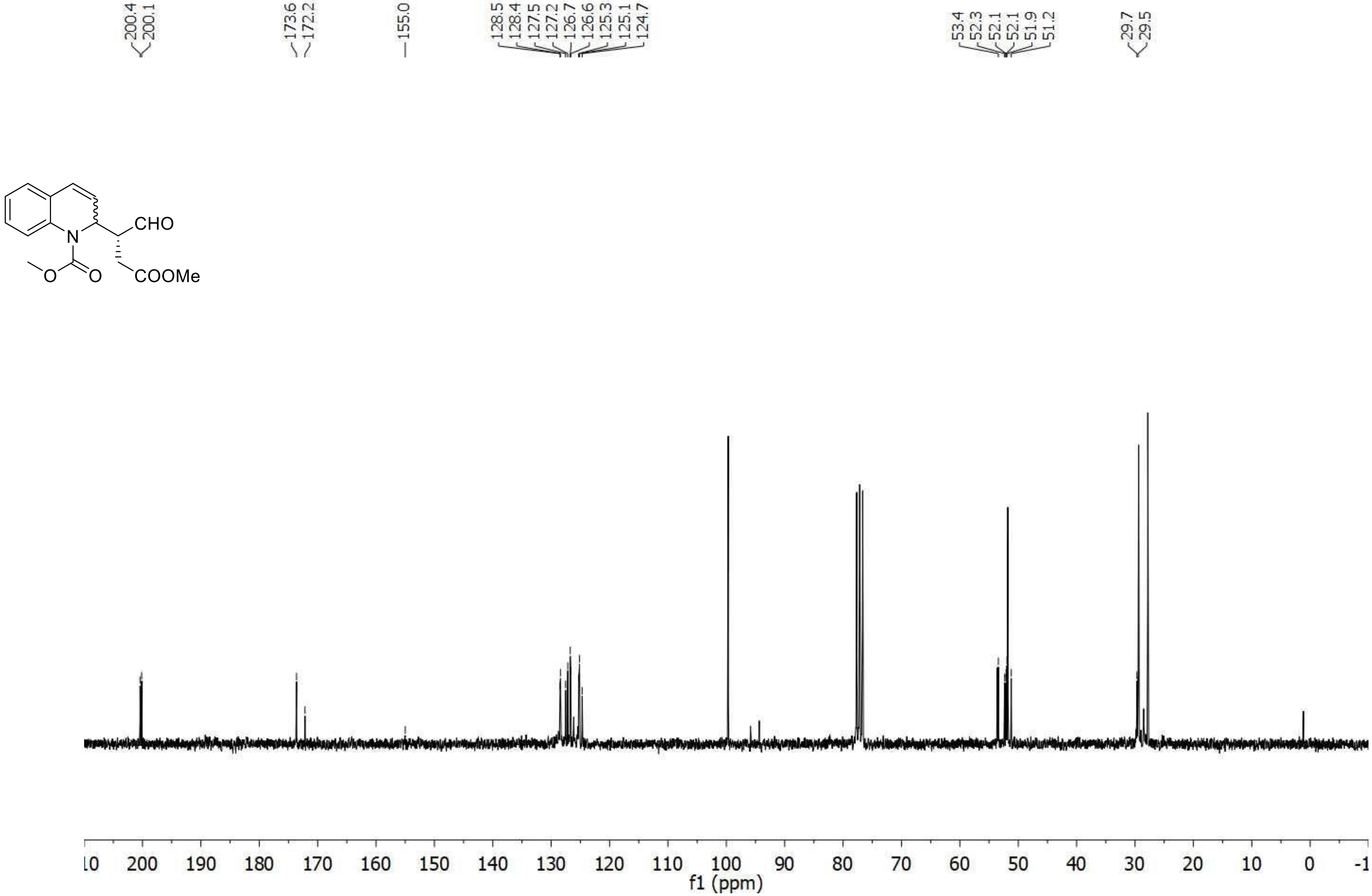

Compound **3bb-*anti*/3bb-*syn*** $^{1}$H NMR (250 MHz, $CD_3Cl$)

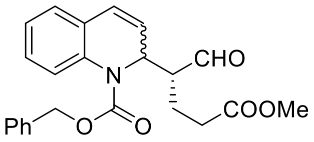


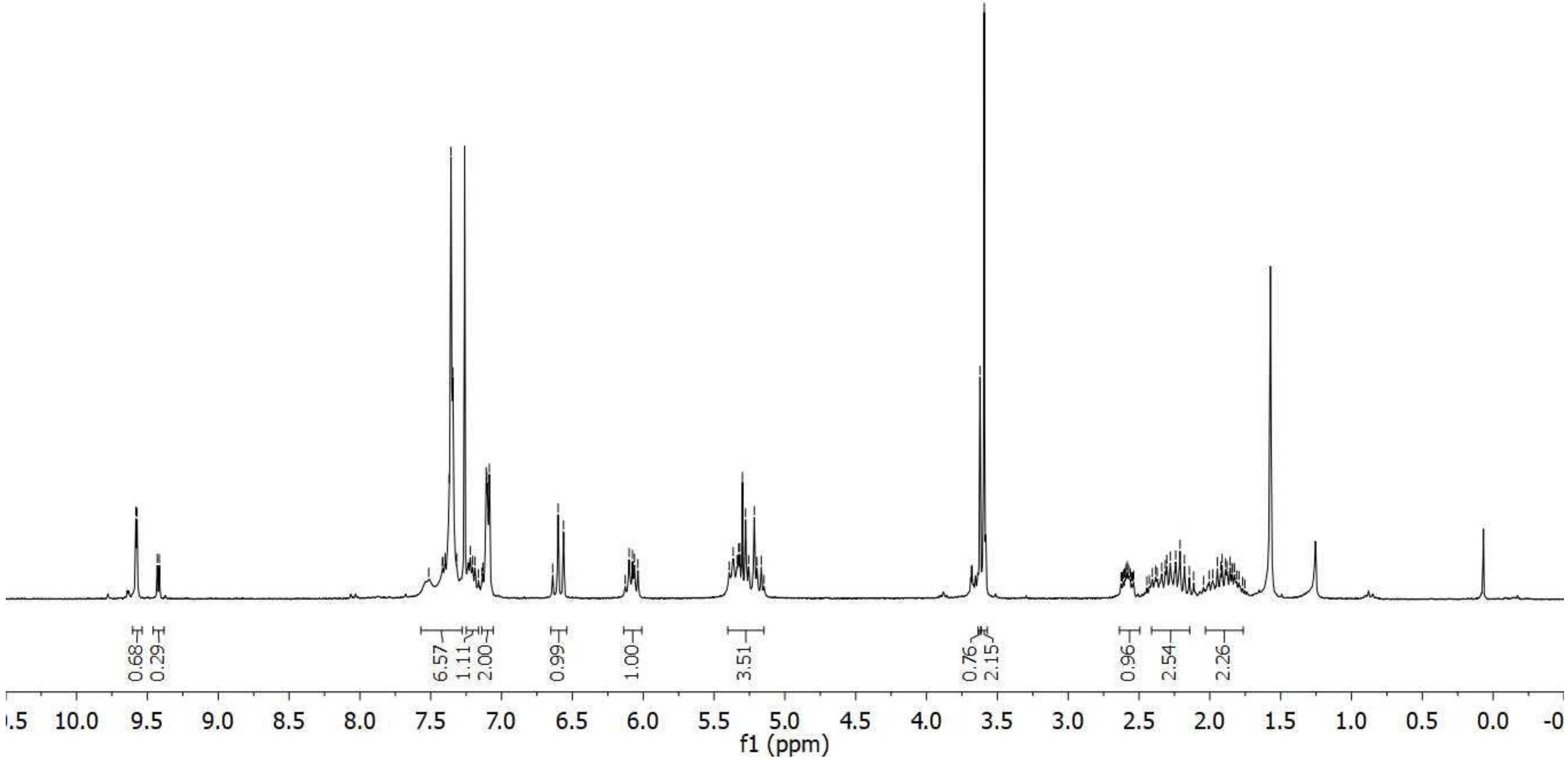

Compound **3bb-*anti*/3bb-*syn*** $^{13}$C NMR (62.5 MHz, CDCl$_3$)

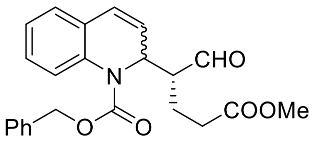


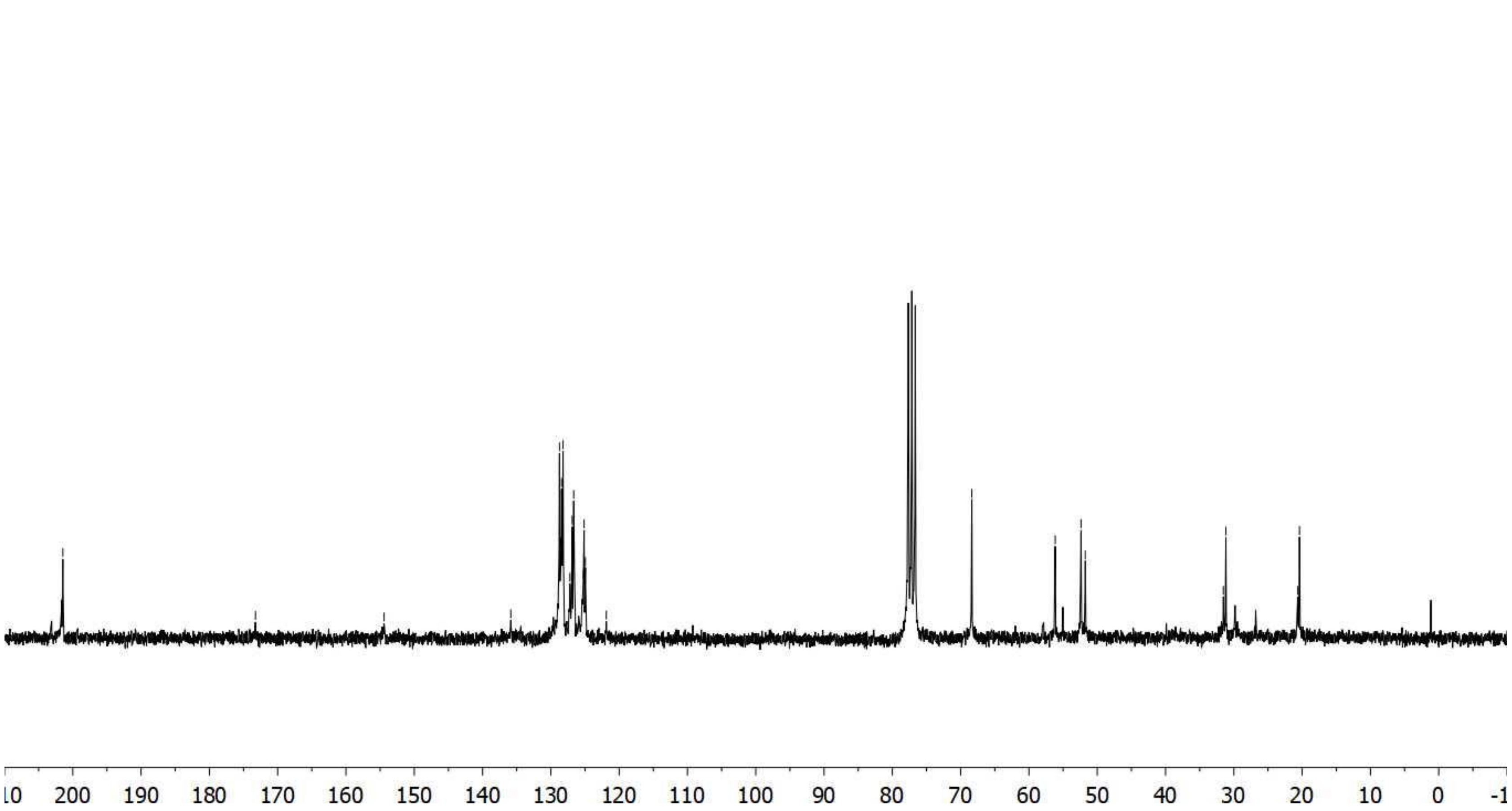

Compound **3ba-*anti*/3ba-*syn*** $^{1}$H NMR (250 MHz, $CD_3Cl$

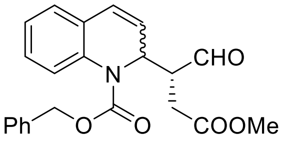


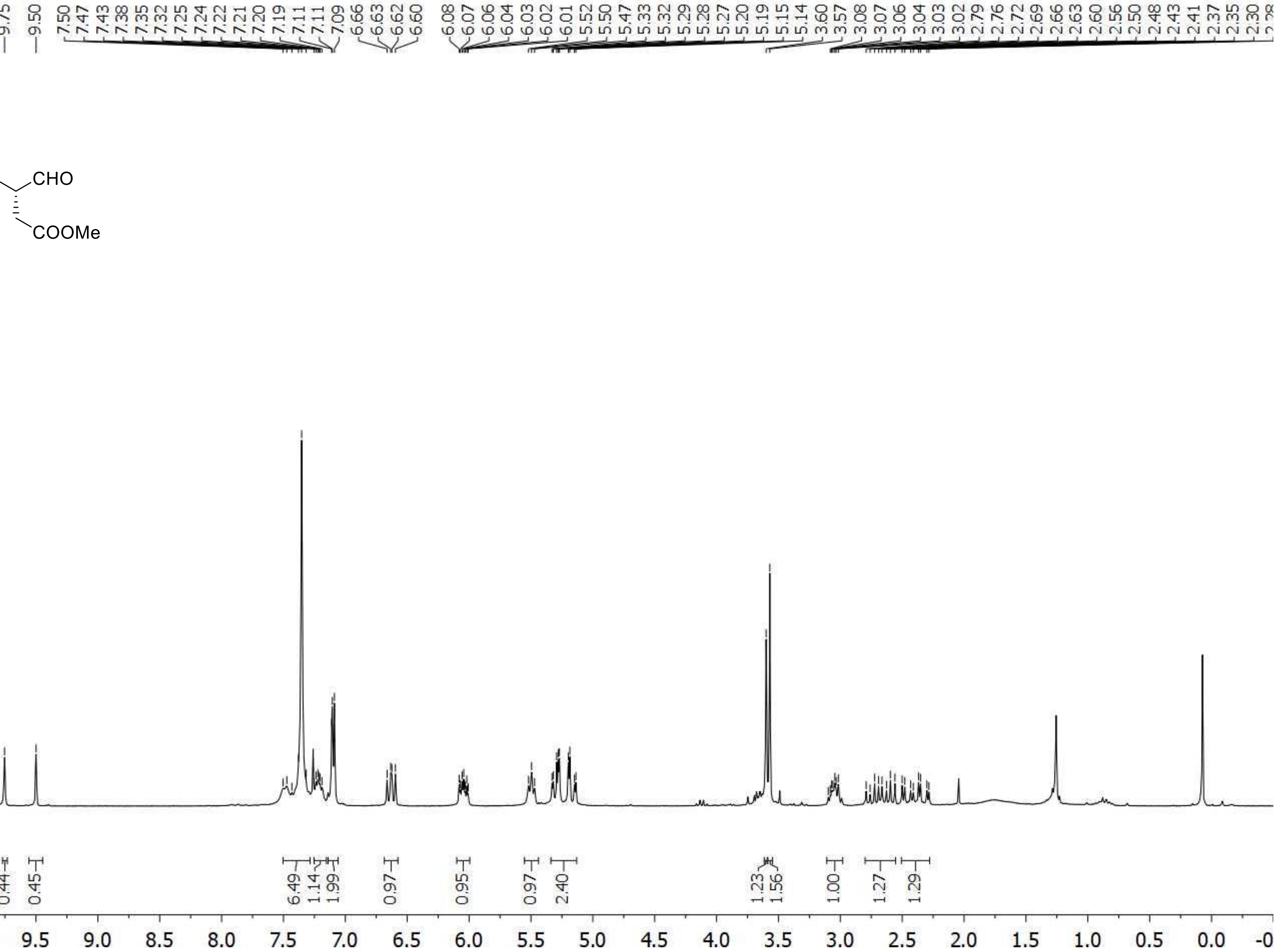

Compound **3ba-*anti*/3ba-*syn*** $^{13}$C NMR (62.5 MHz, $CDCl_3$)

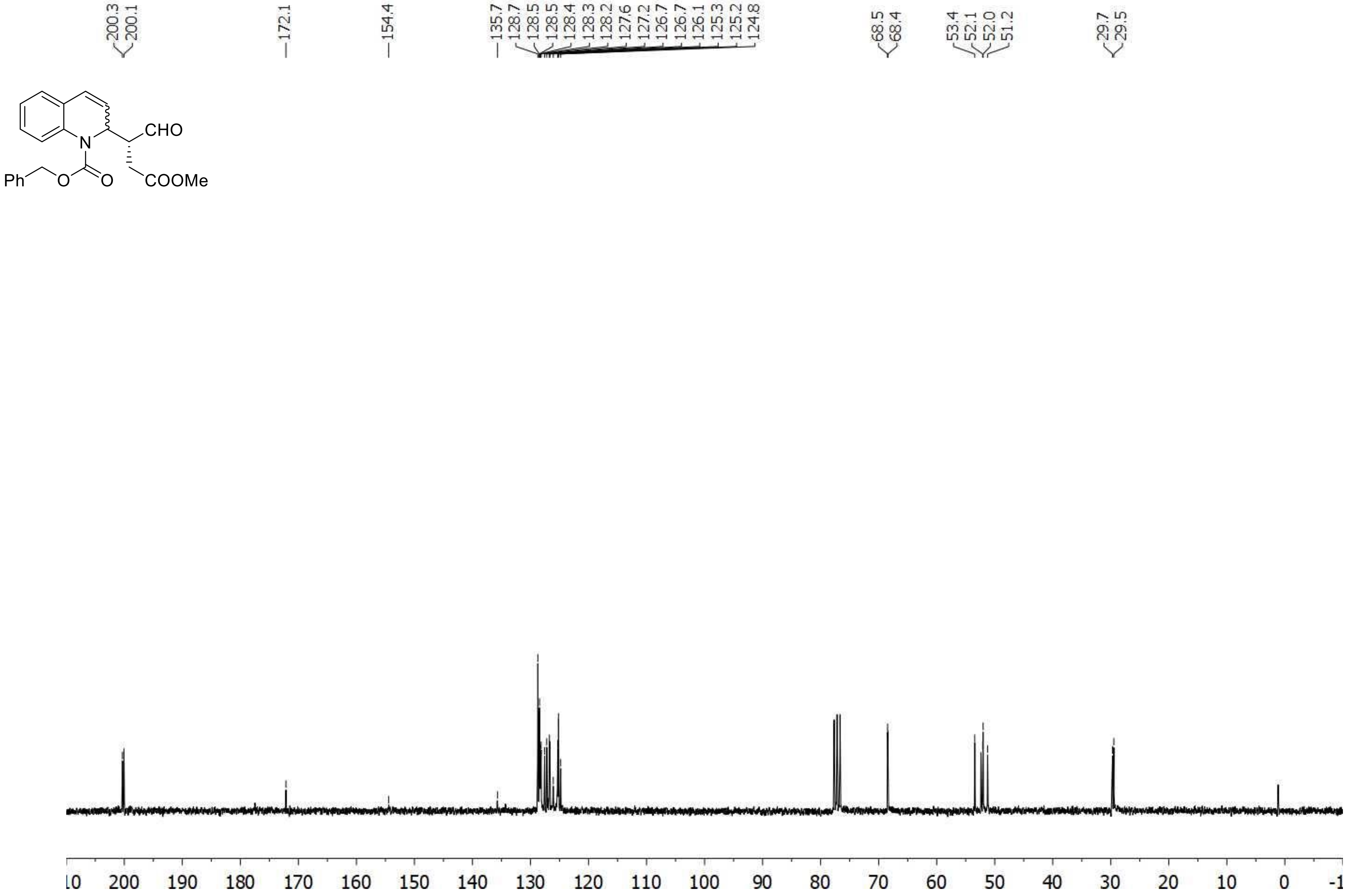

Compound **3cb-*anti*/3cb-*syn*** $^{1}$H NMR (250 MHz, $CD_3Cl$)

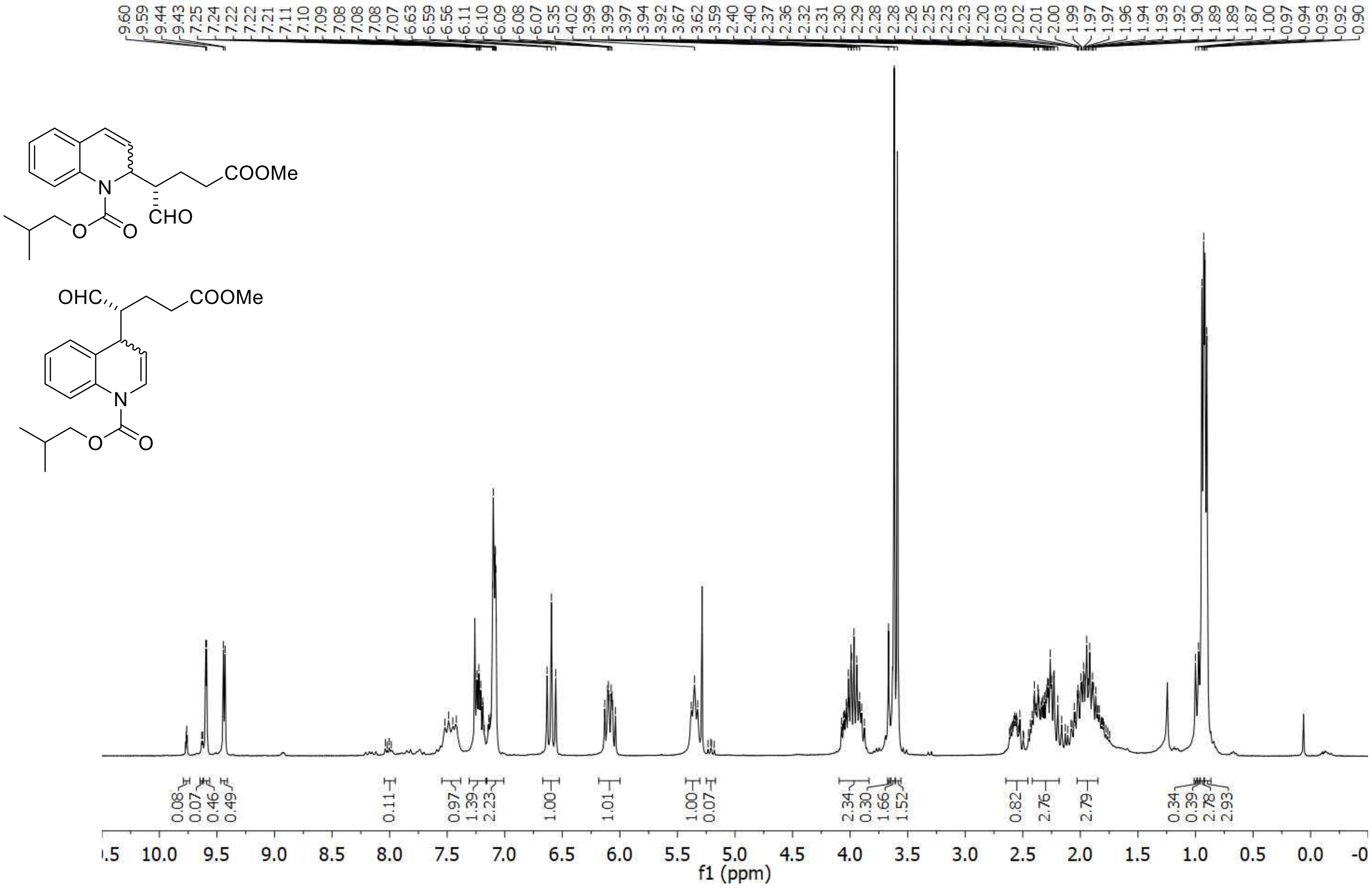

Compound **3cb-*anti*/3cb-*syn*** $^{13}C$ NMR (62.5 MHz, $CD_3Cl$)

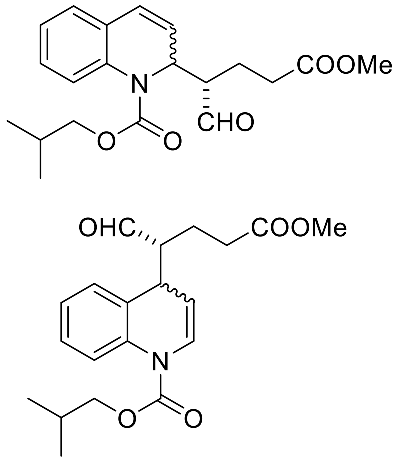


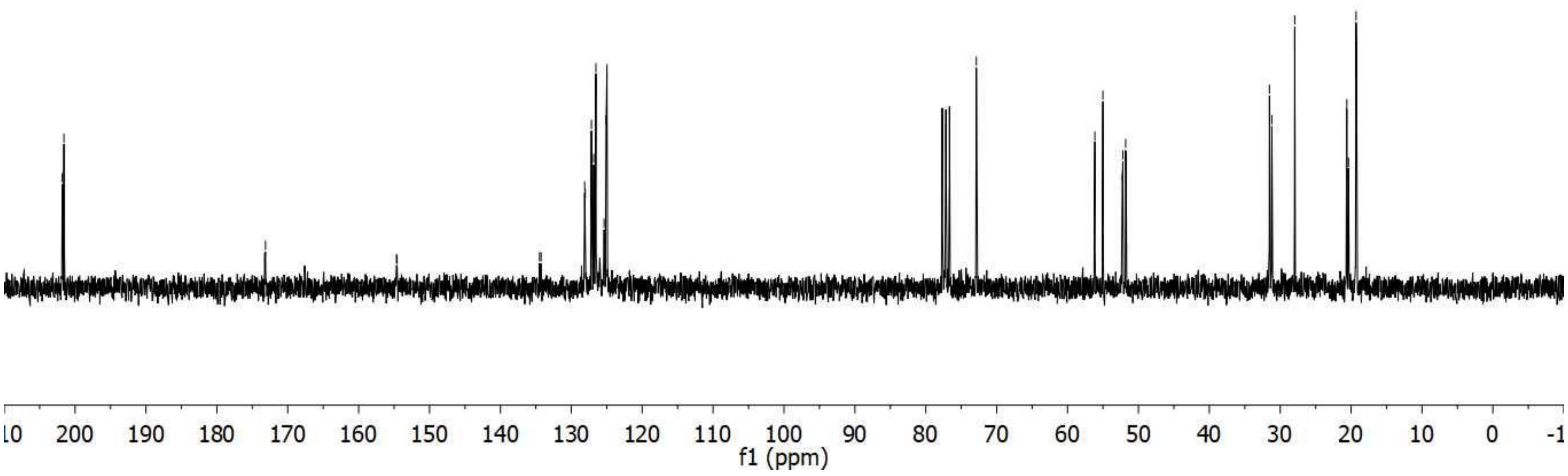

Compound **6b-*anti*/6b-*syn*** $^{1}$H NMR (250 MHz, $CD_3CN$, 65°C)

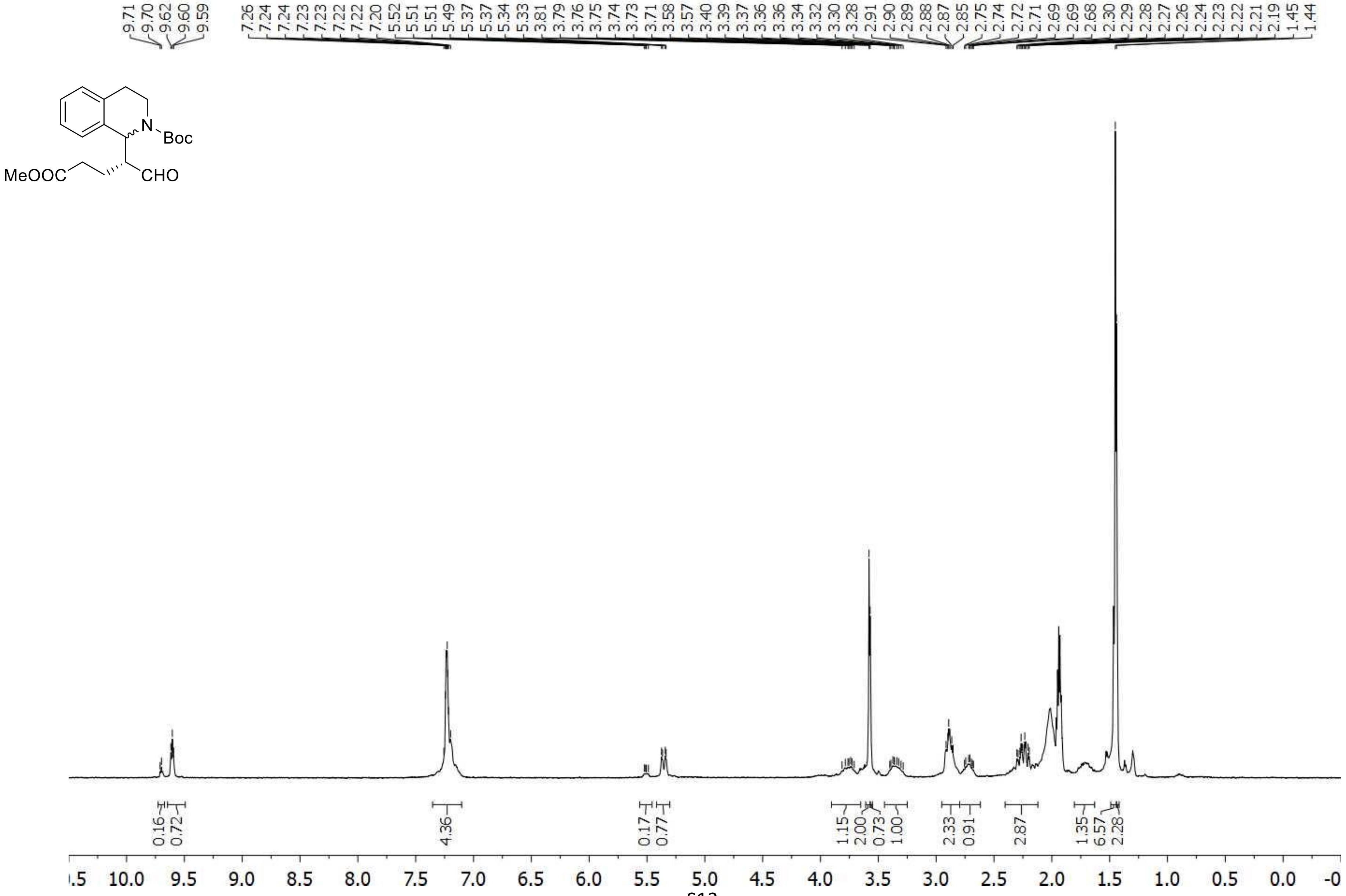

Compound **6b-*anti*/6b-*syn*** $^{13}$C NMR (62.5 MHz, $CDCl_3$)

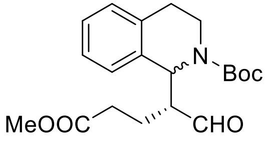


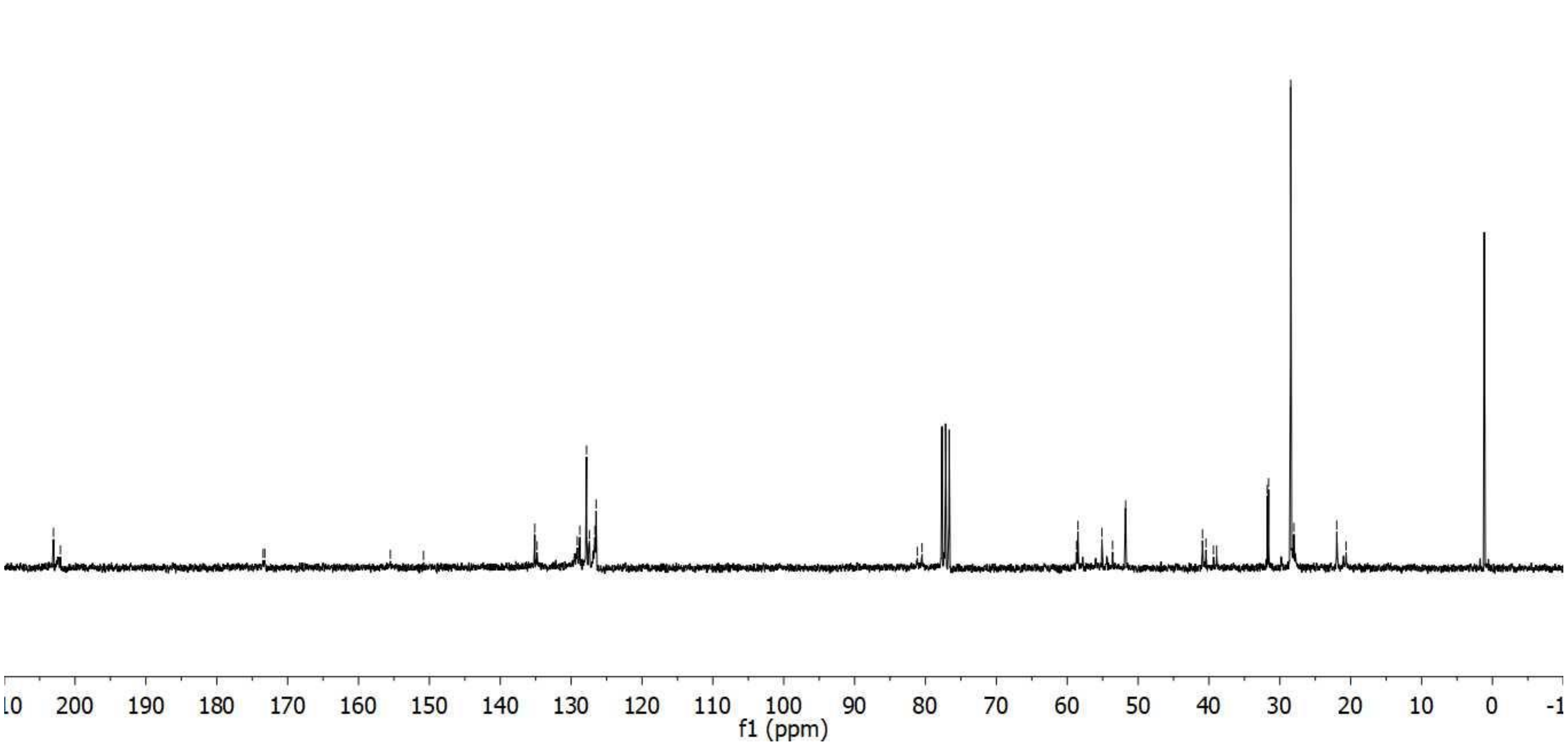

Compound **6a-*anti*/6a-*syn*** $^{1}$H NMR (250 MHz, $CD_3CN$, 65°C)

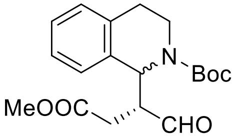


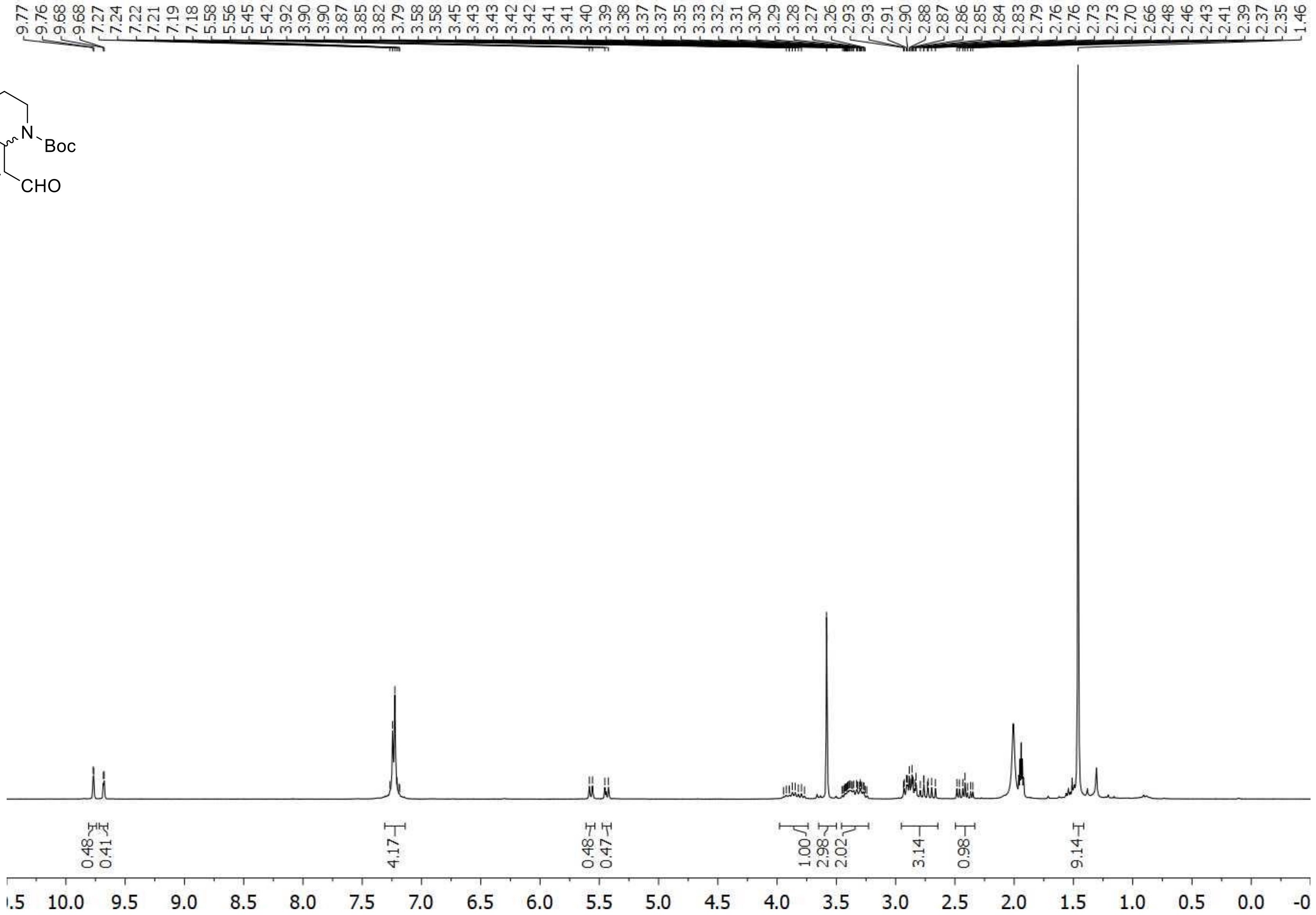

Compound **6a-*anti*/6a-*syn*** $^{13}C$ NMR (62.5 MHz, $CD_3CN$)

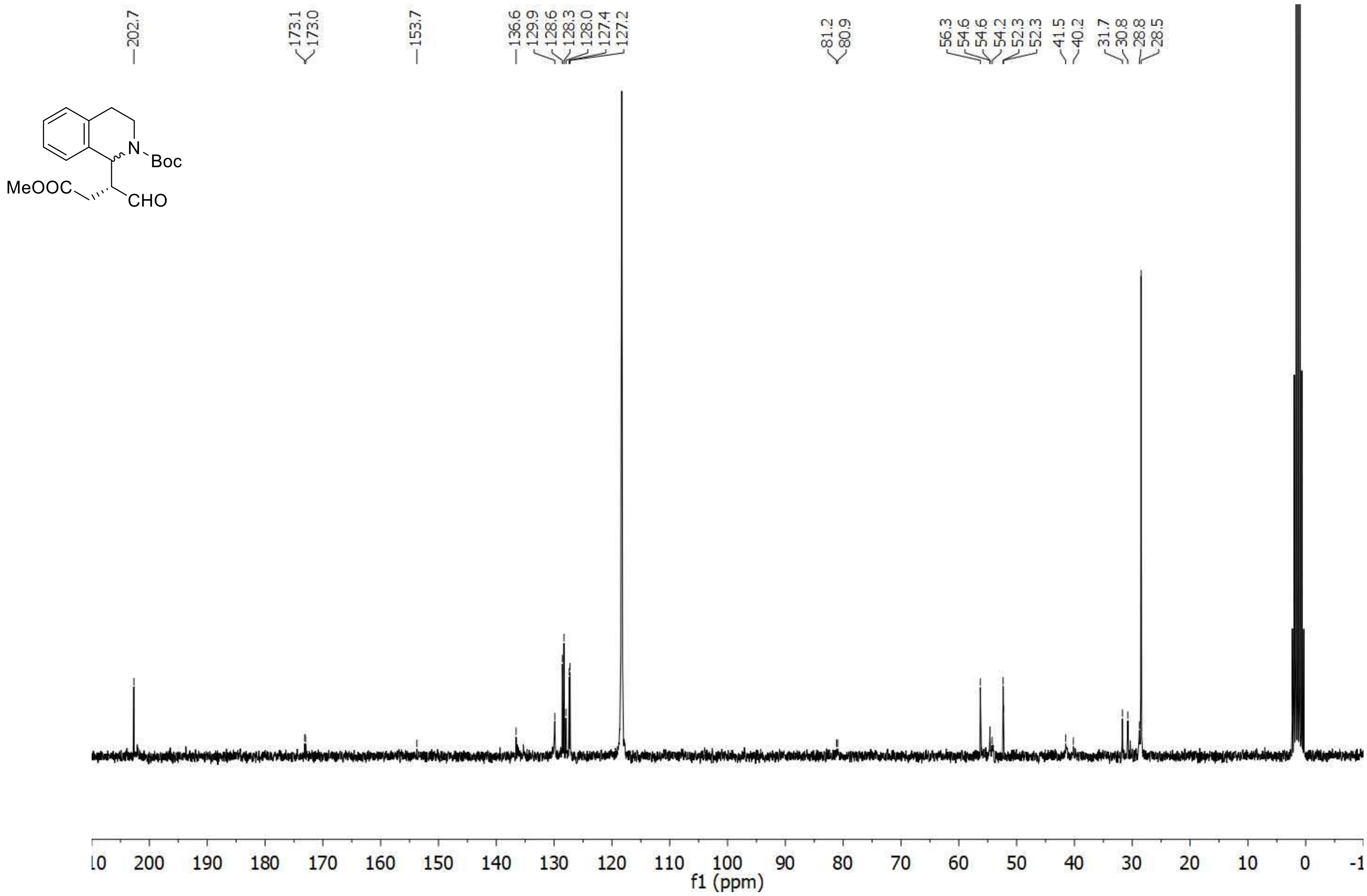

Compound **7a-*anti*/7a-*syn*** $^{1}$H NMR (250 MHz, $CDCl_3$)

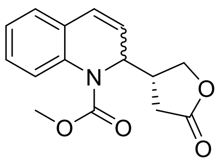

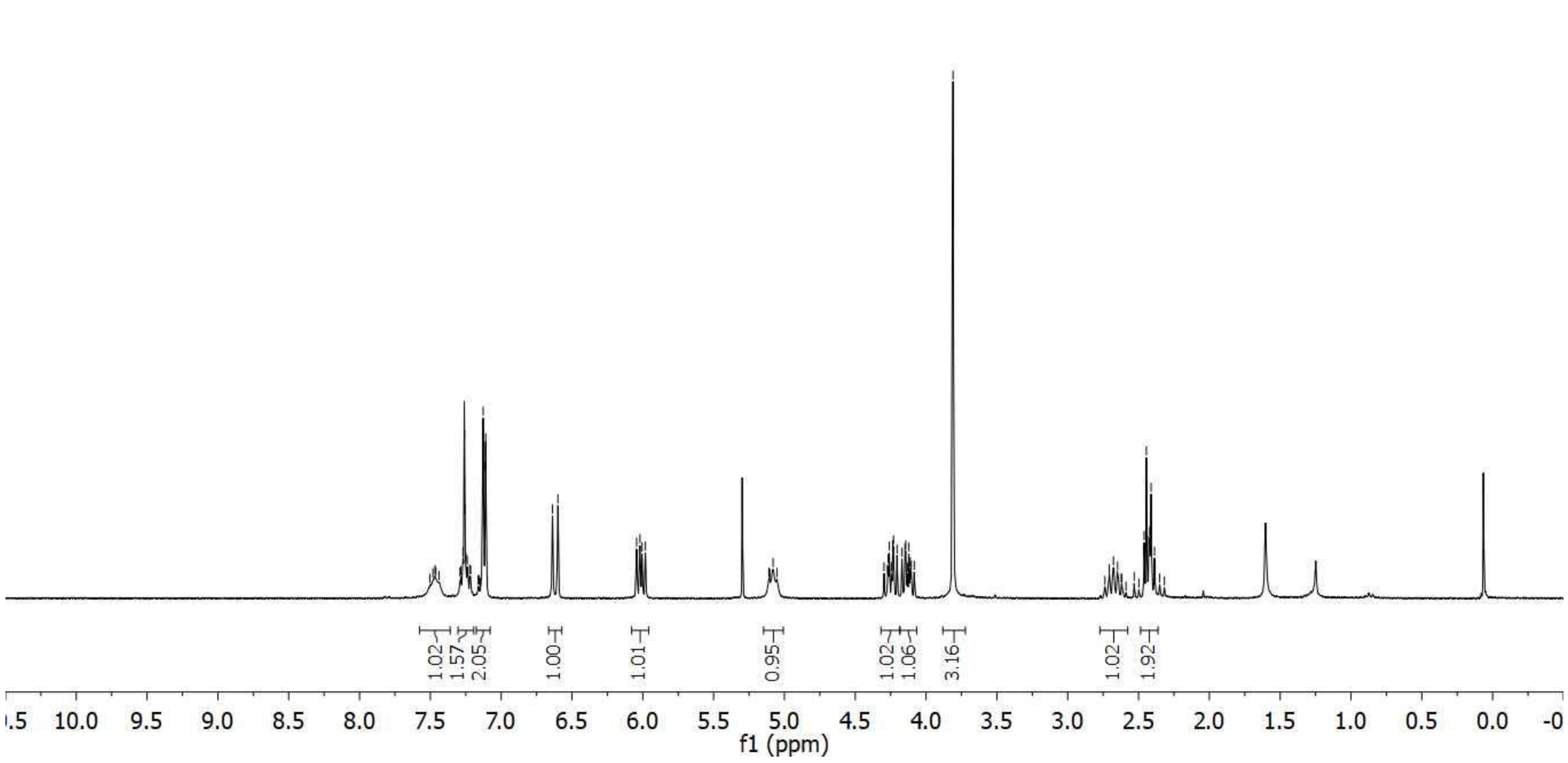

Compound **7a-*anti*/7a-*syn*** $^{13}$C NMR (62.5 MHz, $CDCl_3$)

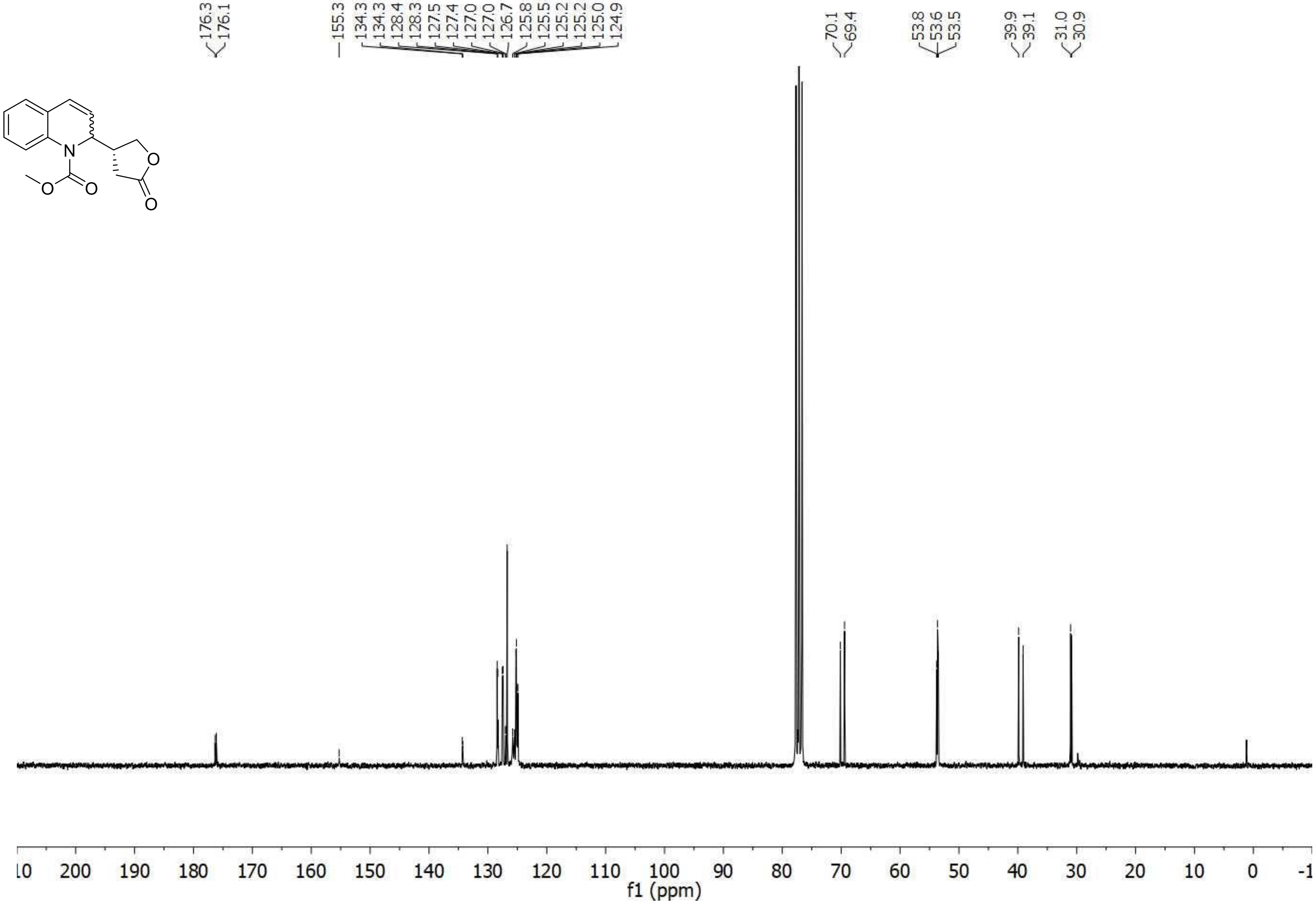

Compound **7b-*anti*/7b-*syn*** $^{1}$H NMR (250 MHz, $CDCl_3$)

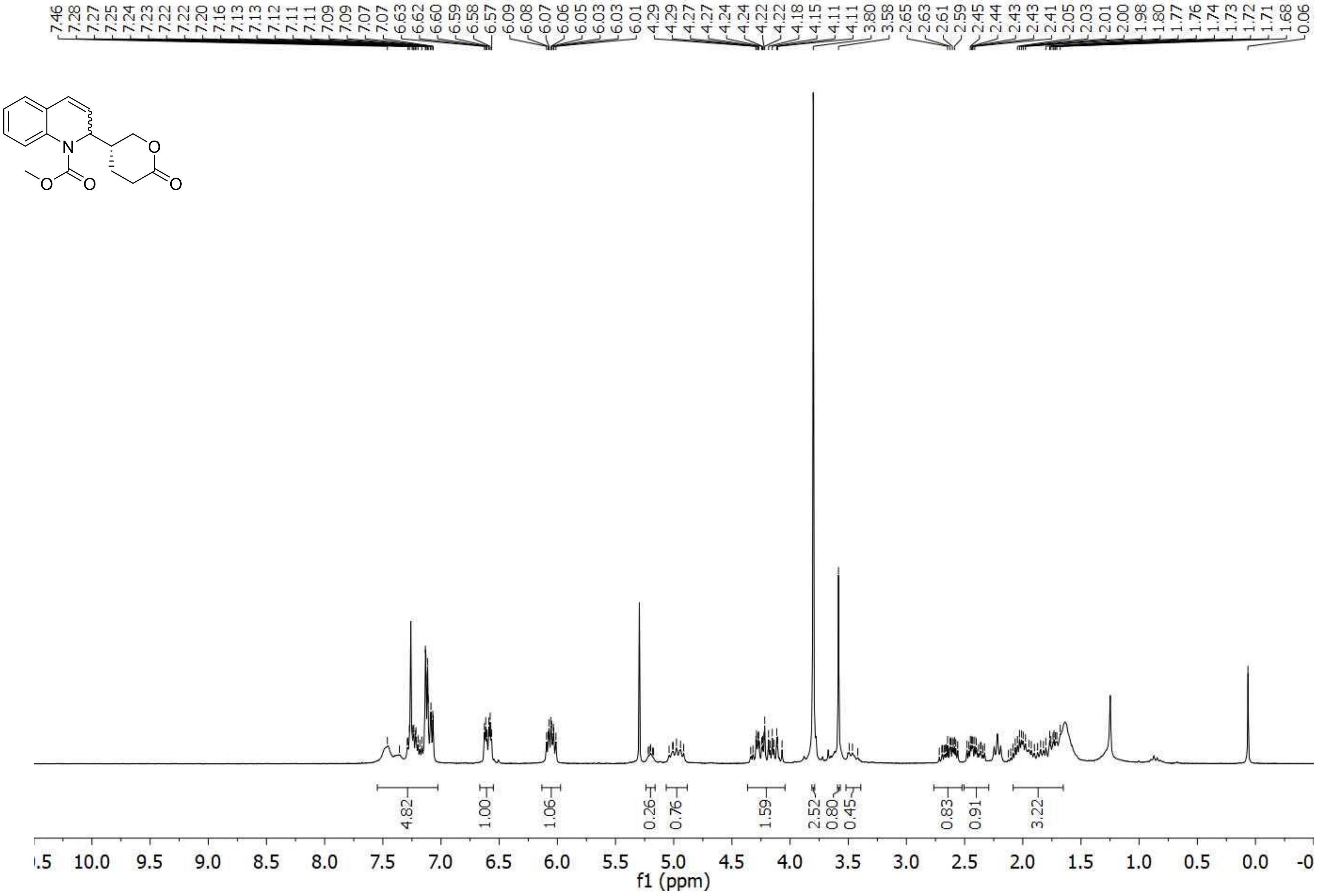

Compound **7b-*anti*/7b-*syn*** $^{13}$C NMR (62.5 MHz CDCl$_3$)

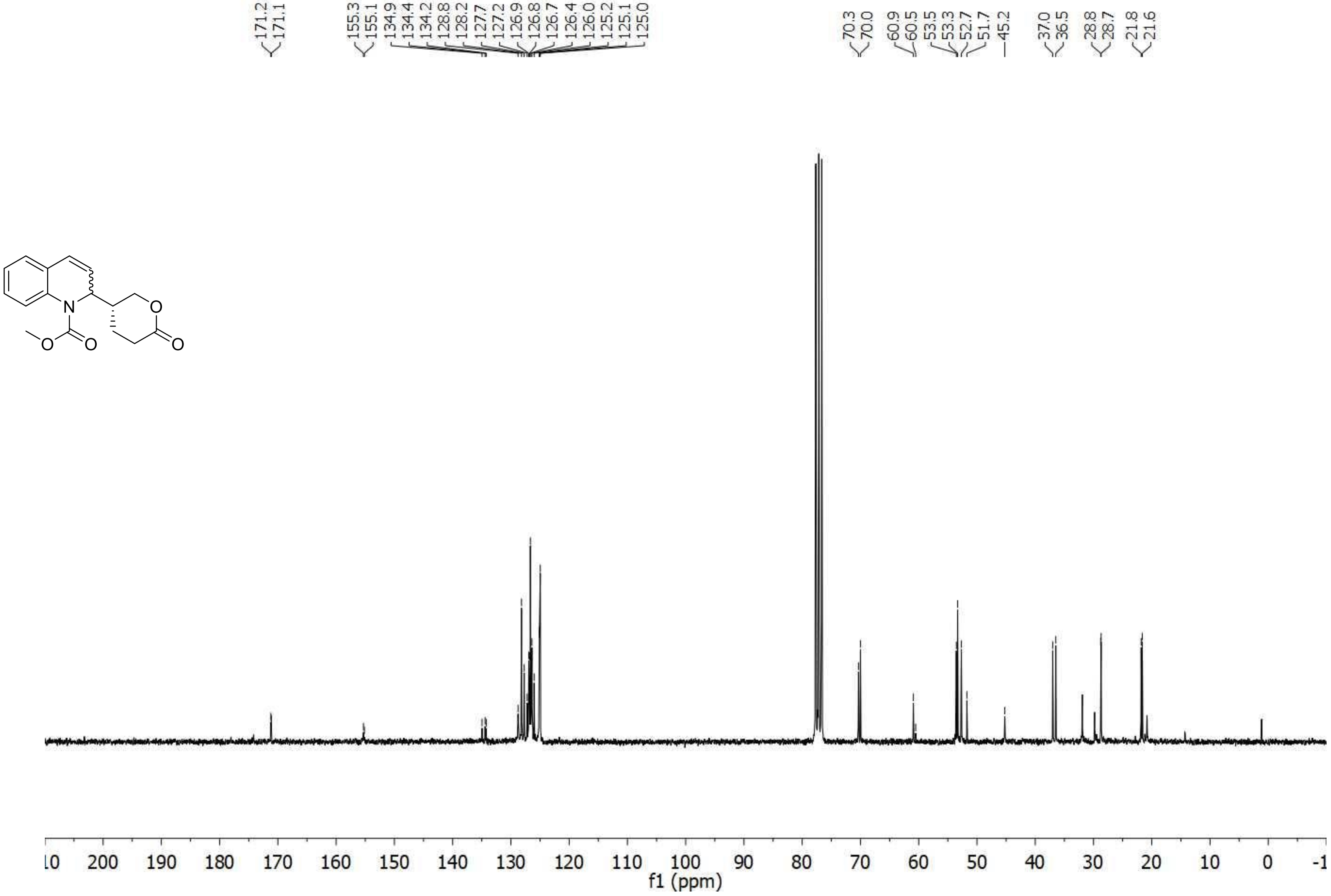

Compound **8-*anti*/8-*syn*** $^{1}$H NMR (250 MHz, $CDCl_3$)

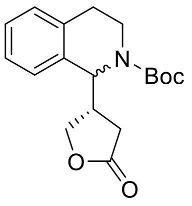

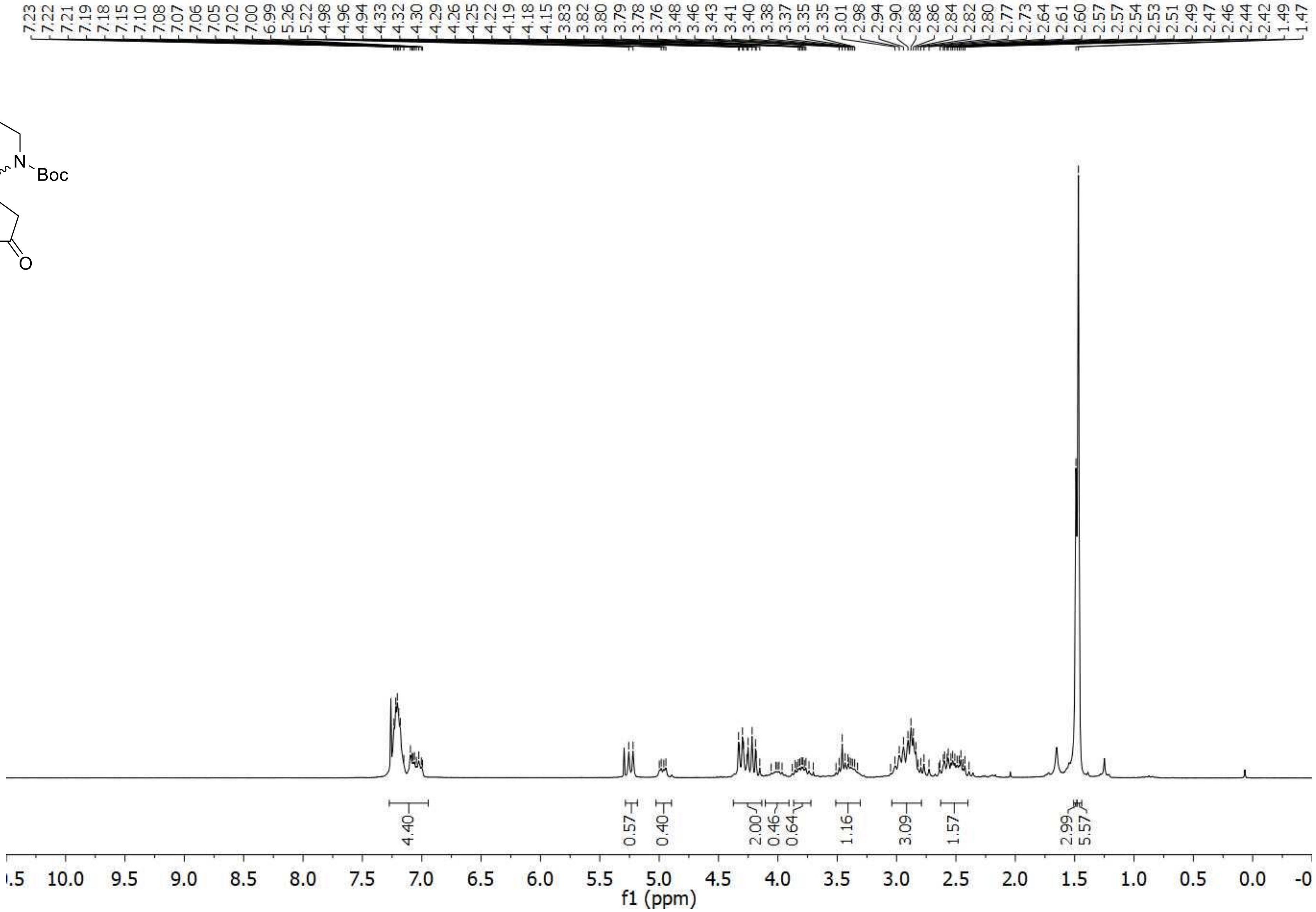

Compound **8-*anti*/8-*syn*** $^{13}$C NMR (62.5 MHz, $CDCl_3$)

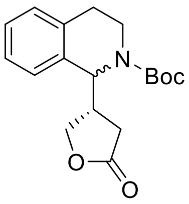


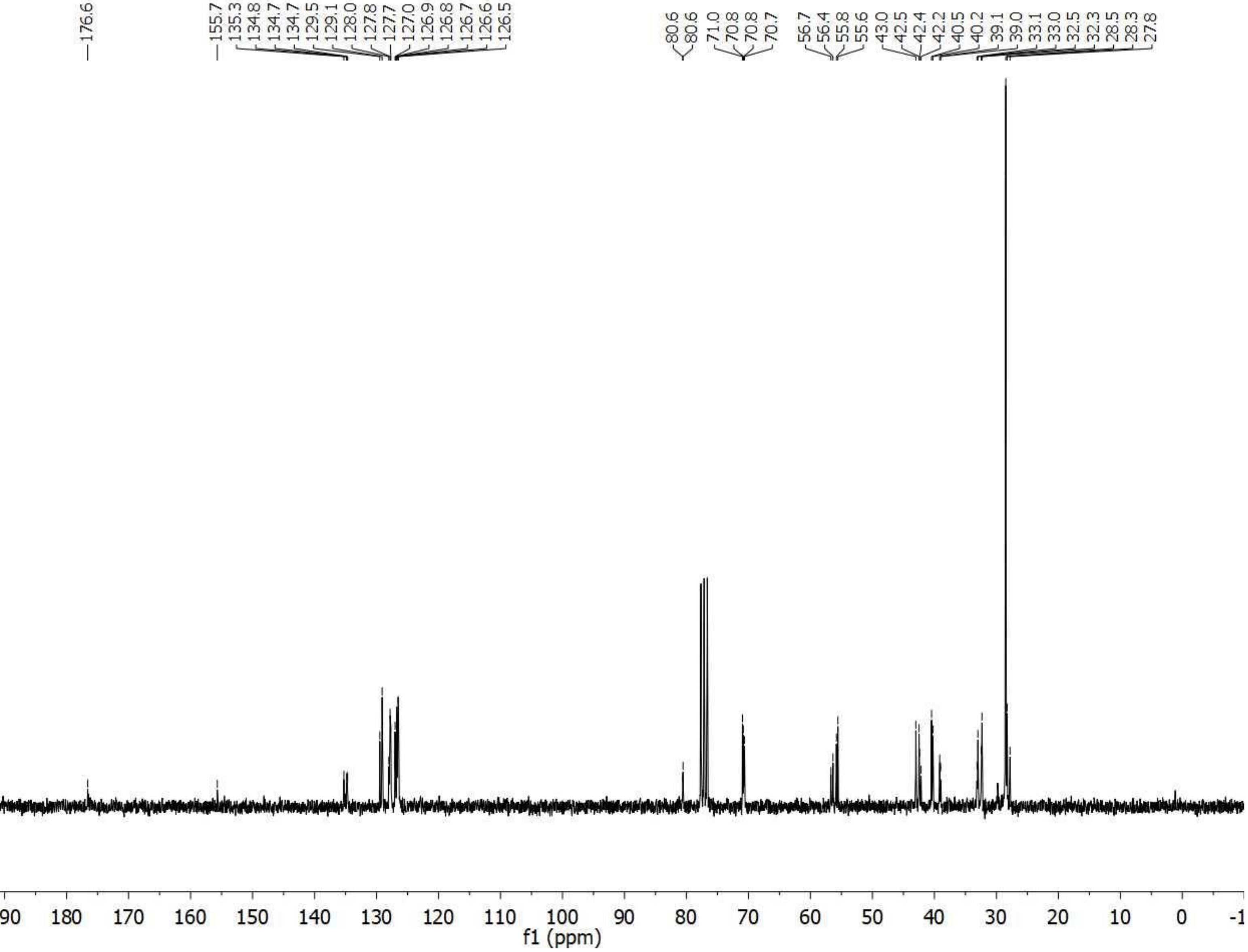

# Computational data

-----------------------------------------------------

**Optimization and Frequency Calculation PW6B95D3/def2-SVP**

------------------------------------------------------

TS-*Si*-*Si*

*CARTESIAN COORDINATES*

| | | | |
|---|---|---|---|
| C | -4.287948 | 1.622694 | 0.829726 |
| C | -3.835830 | 0.881632 | 1.926267 |
| C | -2.937769 | 1.479218 | 2.849331 |
| C | -2.592749 | 2.836118 | 2.689685 |
| C | -3.080779 | 3.567915 | 1.629990 |
| C | -3.911036 | 2.946489 | 0.691008 |
| C | -2.373865 | 0.691623 | 3.890763 |
| C | -2.609003 | -0.653970 | 3.935176 |
| C | -3.457474 | -1.231841 | 2.987770 |
| N | -4.204077 | -0.456500 | 2.157277 |
| C | -5.408032 | -1.090194 | 1.678887 |
| O | -5.568779 | -2.270047 | 1.768002 |
| O | -6.267949 | -0.217992 | 1.222049 |
| C | -7.524448 | -0.750613 | 0.782138 |
| C | -1.630452 | -2.165773 | 1.388158 |
| C | -2.553038 | -2.850636 | 0.421225 |
| C | -1.958502 | -3.021463 | -0.988071 |
| C | -0.589769 | -3.638094 | -0.961072 |
| O | 0.431376 | -3.000492 | -0.850657 |
| C | -1.170961 | -0.899274 | 1.143042 |
| N | -0.194684 | -0.264761 | 1.806328 |
| C | 0.245146 | 1.078206 | 1.478244 |

| | | | |
|---|---|---|---|
| C | 1.001256 | 1.503981 | 2.732111 |
| C | 1.538157 | 0.207894 | 3.346123 |
| C | 0.586757 | -0.889198 | 2.868667 |
| C | 1.109575 | 1.226495 | 0.167558 |
| C | 2.405568 | 0.453643 | 0.347881 |
| C | 2.423975 | -0.941266 | 0.372642 |
| C | 3.593652 | -1.625876 | 0.681642 |
| C | 4.767469 | -0.940469 | 0.971325 |
| C | 4.748394 | 0.447541 | 0.944625 |
| C | 3.581802 | 1.138098 | 0.636087 |
| C | 3.562458 | -3.128758 | 0.684986 |
| F | 3.428874 | -3.625145 | -0.542942 |
| C | 5.971853 | 1.239784 | 1.311746 |
| F | 5.874680 | 1.714734 | 2.556749 |
| C | 0.295300 | 0.804516 | -1.060845 |
| C | -0.856503 | 1.544443 | -1.342760 |
| C | -1.607578 | 1.290384 | -2.484367 |
| C | -1.235417 | 0.281048 | -3.365735 |
| C | -0.098225 | -0.461187 | -3.083445 |
| C | 0.669018 | -0.195004 | -1.955078 |
| C | 0.276410 | -1.595331 | -3.998048 |
| F | -0.555301 | -2.634952 | -3.822955 |
| C | -2.844683 | 2.090236 | -2.769435 |
| F | -2.753949 | 3.332456 | -2.279687 |
| O | 1.337210 | 2.603290 | 0.116510 |
| Si | 1.859092 | 3.656261 | -1.127516 |
| C | 3.286750 | 4.621964 | -0.414357 |
| C | 0.438635 | 4.813556 | -1.478138 |
| C | 2.370628 | 2.690109 | -2.641975 |
| F | -3.929571 | 1.533737 | -2.199049 |
| F | -3.092967 | 2.177677 | -4.068652 |

| | | | |
|---|---|---|---|
| F | 1.510841 | -2.029513 | -3.776801 |
| F | 0.179994 | -1.236366 | -5.276209 |
| F | 2.519643 | -3.582537 | 1.404479 |
| F | 4.666249 | -3.646721 | 1.215664 |
| F | 7.077634 | 0.506402 | 1.243495 |
| F | 6.121922 | 2.293122 | 0.502666 |
| O | -0.611667 | -4.957375 | -1.027802 |
| C | 0.659826 | -5.603172 | -0.991370 |
| H | -0.630995 | 1.714491 | 1.303185 |
| H | -0.087689 | -1.242086 | 3.657806 |
| H | -1.093003 | -2.786858 | 2.104145 |
| H | 3.040620 | 5.002323 | 0.585133 |
| H | 4.211119 | 4.036849 | -0.341024 |
| H | 3.500831 | 5.488508 | -1.053344 |
| H | 0.768648 | 5.627039 | -2.137062 |
| H | -0.405076 | 4.313875 | -1.966388 |
| H | 0.076534 | 5.268781 | -0.547114 |
| H | 2.952446 | 3.341753 | -3.306999 |
| H | 3.007285 | 1.835549 | -2.377216 |
| H | 1.509752 | 2.316172 | -3.208913 |
| H | 1.112800 | -1.772770 | 2.484899 |
| H | -1.144054 | 2.363737 | -0.686395 |
| H | 1.570404 | -0.777281 | -1.811206 |
| H | -1.812291 | 0.091995 | -4.268014 |
| H | 1.784104 | 2.229805 | 2.495875 |
| H | 0.300846 | 1.998401 | 3.416330 |
| H | 2.555749 | 0.005470 | 2.997566 |
| H | 1.581633 | 0.258332 | 4.438142 |
| H | 1.527043 | -1.516977 | 0.156907 |
| H | 3.583252 | 2.222704 | 0.648575 |
| H | 5.679668 | -1.478223 | 1.213933 |

| | | | |
|---|---|---|---|
| H | -3.789889 | -2.263141 | 3.053436 |
| H | -2.157255 | -1.298106 | 4.683859 |
| H | -1.747642 | 1.174008 | 4.638537 |
| H | -4.919707 | 1.170189 | 0.078133 |
| H | -8.031902 | -1.239602 | 1.618584 |
| H | -7.362226 | -1.476927 | -0.019580 |
| H | -8.098565 | 0.104670 | 0.425436 |
| H | -1.922024 | 3.290025 | 3.416588 |
| H | -4.263674 | 3.501854 | -0.173983 |
| H | -2.808017 | 4.613650 | 1.511024 |
| H | -1.640932 | -0.307968 | 0.356360 |
| H | -3.484974 | -2.284156 | 0.300581 |
| H | -2.843962 | -3.829270 | 0.817746 |
| H | -2.628781 | -3.632279 | -1.601090 |
| H | -1.850964 | -2.038929 | -1.460190 |
| H | 0.460984 | -6.671252 | -1.088773 |
| H | 1.287101 | -5.246685 | -1.814851 |
| H | 1.171447 | -5.384390 | -0.048895 |

Electronic Energy = -3561.94087506 (Hartree/Particle)

Dipole Moment (Debye): 14.1936

index 0

Harmonic frequencies

Number of imaginary frequencies= 1

Negatives Eigenvalues: -44.717

*THERMOCHEMICAL DATA*

Temperature 298.150 Kelvin. Pressure 1.00000 Atm.

Zero-point correction= 0.782017 (Hartree/Particle)

Thermal correction to Energy= 0.839657

Thermal correction to Enthalpy= 0.840601

Thermal correction to Gibbs Free Energy= 0.688946

Sum of electronic and zero-point Energies= -3561.158858

Sum of electronic and thermal Energies= -3561.101218

Sum of electronic and thermal Enthalpies= -3561.100274

Sum of electronic and thermal Free Energies= -3561.251929

---------------------------------------------------------------

TS-*Re*-*Re*

*CARTESIAN COORDINATES*

| | | | |
|---|---|---|---|
| C | -4.142911 | 2.497929 | 1.030378 |
| C | -2.764786 | 2.556546 | 0.866540 |
| C | -2.035924 | 1.428301 | 0.509958 |
| C | -2.676726 | 0.210501 | 0.289218 |
| C | -4.055897 | 0.150948 | 0.463565 |
| C | -4.778672 | 1.280518 | 0.831312 |
| C | -1.901433 | -1.065729 | 0.001942 |
| C | -0.865419 | -0.929569 | -1.119755 |
| C | -0.169881 | -2.085155 | -1.477762 |
| C | 0.760013 | -2.076609 | -2.511324 |
| C | 1.035540 | -0.906675 | -3.207091 |
| C | -0.603862 | 0.235986 | -1.837867 |
| C | -1.175379 | -1.556734 | 1.306460 |
| N | -0.013265 | -0.778302 | 1.708733 |
| C | -0.309704 | 0.131396 | 2.819133 |
| C | -1.738047 | -0.221035 | 3.251159 |
| C | -2.053450 | -1.546033 | 2.552070 |
| C | 1.223084 | -1.240006 | 1.485090 |
| C | 2.409584 | -0.669410 | 1.882391 |

| | | | |
|---|---|---|---|
| O | -2.767374 | -2.121939 | -0.283356 |
| Si | -3.625294 | -2.567396 | -1.696308 |
| C | -3.476280 | -1.230452 | -2.995977 |
| C | -5.395496 | -2.810398 | -1.164074 |
| C | -2.907124 | -4.183417 | -2.282880 |
| H | -0.853697 | -2.575097 | 1.066397 |
| H | 0.422536 | -0.060118 | 3.615069 |
| H | 2.375247 | 0.289633 | 2.403184 |
| H | -5.446608 | -3.414226 | -0.249278 |
| H | -5.924219 | -1.866730 | -0.984205 |
| H | -5.946466 | -3.348529 | -1.946187 |
| H | -3.518990 | -4.592578 | -3.097338 |
| H | -1.883237 | -4.080727 | -2.659669 |
| H | -2.900830 | -4.921862 | -1.471124 |
| H | -4.246643 | -1.387237 | -3.762480 |
| H | -3.633697 | -0.230188 | -2.571393 |
| H | -2.501378 | -1.244467 | -3.497427 |
| H | -0.200934 | 1.180595 | 2.519504 |
| H | -0.383700 | -3.020752 | -0.964143 |
| H | -1.141817 | 1.154611 | -1.634610 |
| H | 1.766140 | -0.896177 | -4.011852 |
| H | -3.112422 | -1.670723 | 2.307190 |
| H | -1.761278 | -2.392680 | 3.183700 |
| H | -2.442017 | 0.560485 | 2.951430 |
| H | -1.808766 | -0.310222 | 4.339166 |
| H | -0.957697 | 1.508205 | 0.424954 |
| H | -4.570009 | -0.795526 | 0.332372 |
| H | -4.710198 | 3.381475 | 1.308340 |
| C | 2.852685 | 0.218459 | -0.342301 |
| N | 3.273762 | 1.485053 | -0.070511 |
| H | 1.791925 | 0.135264 | -0.535601 |

| | | | |
|---|---|---|---|
| C | 3.768206 | -0.738836 | -0.819987 |
| C | 2.210192 | 2.435248 | 0.011078 |
| C | 4.638307 | 1.784811 | 0.081358 |
| C | 5.094445 | -0.439021 | -0.852406 |
| H | 3.387078 | -1.701969 | -1.142408 |
| O | 1.092477 | 2.084336 | 0.269418 |
| O | 2.600301 | 3.643323 | -0.281024 |
| C | 5.569337 | 0.811350 | -0.357222 |
| C | 5.104026 | 2.963553 | 0.673739 |
| H | 5.815699 | -1.158235 | -1.235832 |
| C | 1.571830 | 4.639787 | -0.353248 |
| C | 6.945846 | 1.078021 | -0.250394 |
| C | 6.464950 | 3.187024 | 0.779126 |
| H | 4.412952 | 3.703280 | 1.055581 |
| H | 1.033725 | 4.693215 | 0.596481 |
| H | 0.873149 | 4.379877 | -1.153243 |
| H | 2.085074 | 5.575961 | -0.573203 |
| C | 7.394142 | 2.255973 | 0.304603 |
| H | 7.649040 | 0.325524 | -0.601156 |
| H | 6.811197 | 4.106939 | 1.244942 |
| H | 8.459555 | 2.455039 | 0.388079 |
| H | 1.268184 | -2.185218 | 0.941691 |
| C | 0.350923 | 0.248192 | -2.849028 |
| C | 3.631394 | -1.514707 | 2.076297 |
| H | 4.539720 | -0.933412 | 1.872200 |
| H | 3.617792 | -2.349122 | 1.363693 |
| C | 3.733855 | -2.090461 | 3.484940 |
| H | 3.704251 | -1.309234 | 4.254420 |
| H | 4.695531 | -2.604632 | 3.622855 |
| C | 2.652128 | -3.095092 | 3.783192 |
| O | 1.918416 | -3.585640 | 2.960820 |

| | | | |
|---|---|---|---|
| O | 2.606460 | -3.396822 | 5.074363 |
| C | 1.653813 | -4.379236 | 5.459003 |
| H | 1.838315 | -5.321283 | 4.931915 |
| H | 1.769922 | -4.514148 | 6.535507 |
| H | 0.638160 | -4.042235 | 5.223610 |
| C | 0.735831 | 1.553837 | -3.480626 |
| C | 1.496848 | -3.348832 | -2.820995 |
| C | -2.022581 | 3.846573 | 1.043091 |
| C | -6.261722 | 1.140823 | 1.037760 |
| F | -2.795015 | 4.819926 | 1.506339 |
| F | -0.990404 | 3.706738 | 1.892084 |
| F | -1.499210 | 4.260894 | -0.119973 |
| F | -6.856885 | 2.321535 | 1.171725 |
| F | -6.828582 | 0.506975 | 0.006340 |
| F | -6.524951 | 0.422165 | 2.130629 |
| F | 1.815053 | 2.066107 | -2.844291 |
| F | 1.071885 | 1.418015 | -4.755413 |
| F | -0.227702 | 2.465241 | -3.388255 |
| F | 2.213987 | -3.254745 | -3.933420 |
| F | 2.347942 | -3.648896 | -1.819265 |
| F | 0.668068 | -4.382037 | -2.939269 |

Electronic Energy = -3561.93731011 (Hartree/Particle)

Dipole Moment (Debye): 11.5099

index 1

Number of imaginary frequencies= 1

Negatives Eigenvalues: -110.270

*THERMOCHEMICAL DATA*

Temperature 298.150 Kelvin. Pressure 1.00000 Atm.

Zero-point correction= 0.781646 (Hartree/Particle)

Thermal correction to Energy= 0.839700

Thermal correction to Enthalpy= 0.840644

Thermal correction to Gibbs Free Energy= 0.685624

Sum of electronic and zero-point Energies= -3561.155664

Sum of electronic and thermal Energies= -3561.097610

Sum of electronic and thermal Enthalpies= -3561.096666

Sum of electronic and thermal Free Energies= -3561.251686

---------------------------------------

**Electronic and Free Energies correction**

---------------------------------------

TS-*Si*-*Si*

E(RPW6B95D3/def2-svp) = -3561.94087506

Sum of electronic and thermal Free Energies= -3561.251929

(DG-DE=0.68562411)

RI-MP2-SCS/def2-TZVP -3553.891083453070

Free Energy corrected in vacuo:

-3553.891083453070+0.68894606 = -3553.202137393070

Contribute of solvent (tolune)

Single point E(RPW6B95D3/def2-TZVP) CPCM = -3565.79354184

Single point E(RPW6B95D3/def2-TZVP) SMD = -3565.81694324

---------------------------------------------------------

TS-*Re*-*Re*

E(RPW6B95D3/def2-svp) =  -3561.93731011

Sum of electronic and thermal Free Energies = -3561.251686
(DG-DE=0.68562411)

RI-MP2-SCS/def2-TZVP -3553.889257294106

Free Energy corrected in vacuo:

-3553.889257294106+0.68562411 = -3553.203633184106

Contribute of solvent (tolune)

Single point E(RPW6B95D3/def2-TZVP) CPCM = -3565.78908261
Single point E(RPW6B95D3/def2-TZVP) SMD =  -3565.81371578

---------------------------------------------------

G(TS-*Si*-*Si*)-G(TS-*Re*-*Re*) and corresponidng *Si*-*Si*/*Re*-*Re*

---------------------------------------------------

ΔΔG* (Vacuum)       = -3.9 kJ/mol; *Si*-*Si*/*Re*-*Re* = 17/83
ΔΔG* (Toluene;CPCM) = +3.2 kJ/mol; *Si*-*Si*/*Re*-*Re* = 78/22
ΔΔG* (Toluene;SMD)  = -0.2 kJ/mol; *Si*-*Si*/*Re*-*Re* = 48/51

------------------------------------------------------
